# Historical *δ*^15^N records of *Saccharina* specimens from oligotrophic waters of Japan Sea (Hokkaido)

**DOI:** 10.1371/journal.pone.0180760

**Published:** 2017-07-12

**Authors:** Takanori Kuribayashi, Tsuyoshi Abe, Shigeru Montani

**Affiliations:** 1 Division of Biosphere Science, Graduate School of Environmental Science, Hokkaido University, Sapporo, Hokkaido, Japan; 2 Section of Fisheries Research, Hokkaido Nuclear Energy Environmental Research Center, Kyowa, Iwanai, Hokkaido, Japan; 3 The Hokkaido University Museum, Sapporo, Hokkaido, Japan; 4 Department of Natural History Sciences, Graduate School of Science, Hokkaido University, Sapporo, Hokkaido, Japan; 5 Division of Marine Bioresource and Environmental Science, Graduate School of Fisheries Sciences, Hokkaido University, Hakodate, Hokkaido, Japan; University of Shiga Prefecture, JAPAN

## Abstract

Historically *Saccharina* spp. beds occurred along the west coast of Hokkaido, an oligotrophic area, and were commercially exploited. Currently extensive commercial *Saccharina* spp. beds do not form due to nutrient limitations. Here, we postulate that nutrients assimilated by paleo-*Saccharina* spp. beds may have been derived from spawning herrings (*Clupea pallasii*) acting as organisms that formed a vector from their feeding grounds (Okhotsk Sea and Pacific Ocean) to their spawning area (west coast of Hokkaido, Japan Sea). To test this hypothesis we examined stable nitrogen isotope ratios (*δ*^15^N) of 100– to 135–year-old *Saccharina* specimens preserved at the Herbarium (Hokkaido University Museum). *δ*^15^N values of the paleo-*Saccharina* specimens collected from this region were in the range of 10‰, which is significantly higher than the current 3–7‰ in freshly sampled *Saccharina* spp. This high *δ*^15^N indicates that spawning herring (*Clupea pallasii*) had potentially been a significant source of dissolved inorganic nitrogen (DIN) absorbed by *Saccharina*, acting as an organism forming a vector for transporting nutrients from eutrophic to oligotrophic coastal ecosystems. Our findings support the hypothesis of so-called “herring-derived nutrients.”

## Introduction

The west coast of the Japan Sea off Hokkaido, Japan is affected by the northeastward-flowing Tsushima Warm Current that is characterized by oligotrophy compared with the other coasts (Okhotsk Sea and Pacific coast) of Hokkaido. This oligotrophic condition is one of the causes behind “*isoyake*” namely, the recent decrease or disappearance of kelp resulting in the establishment of poorly vegetated areas [1,2]. On the other hand, before the early 20^th^ century, large-scale *Saccharina* beds had formed [3], and large numbers of herring were caught along the west coast of the Japan Sea off Hokkaido [4]. Because of the high biological productivity, large quantities of nutrients were expected to have been available in this region. However, no scientific data were available to describe the previous levels of nutrients present in this region.

To overcome this limitation, we previously developed a technique involving analysis of stable nitrogen isotope ratios (*δ*^15^N) in the tissue of macroalgal specimens. There are two naturally occurring atomic forms of nitrogen. The common form that contains seven protons and seven neutrons is referred to as nitrogen 14 and expressed as ^14^N. A heavier form that contains an extra neutron is called nitrogen 15, expressed as ^15^N. Stable isotopes of light elements such as ^14^N and ^15^N, which exhibit different reactivities because of their mass differences, result in isotopic fractionation through a variety of physical and chemical processes. The various sources of nitrogen in the ecosystems often have been characterized by ^15^N: ^14^N (expressed as *δ*^15^N) values [5–8]. Since the late 1990s, the *δ*^15^N signature in seaweeds has often been used as an indicator of DIN sources, such as anthropogenic inputs of sewage effluent into the coastal ecosystems [9,10]. That is, algal species synthesize components in the tissue by assimilating nutrients from the surrounding water. This property indicates that the *δ*^15^N value in algal tissue is a reflection of the in situ water column dissolved inorganic nitrogen (DIN) history, and provides temporally integrated information on the biologically available form in marine ecosystems [11]. Water quality parameters, e.g., *δ*^15^N-DIN, DIN concentration, etc. are useful for determining the flows, pulses, and physical distributions of non-continuous (snapshot) values, “snapshots”. However, temporal and spatial variability confounds interpretation of these parameters, and only water quality parameters provide minimal insight into the averaged information on the biological uptake and impact of nutrients [12].

Many kinds of organisms collected in the past have often been preserved in museums as biological specimen collections. Algal specimens have often been preserved there with detailed information regarding the collection area, date, and species name; therefore, analyzing *δ*^15^N in algal specimens is expected to provide information regarding previous DIN status in the collection area. Previous studies have proposed that the *δ*^15^N records in specimens could be useful for tracing and understanding historical environmental change [13–15].

In this study, we compared the present and previous nutrient conditions on the west coast of the Japan Sea off Hokkaido and other coasts, i.e., the Pacific Ocean and Okhotsk Sea off Hokkaido, by examining *δ*^15^N which was used as an indicator of the DIN status in algal tissue of *Saccharina* specimens and freshly collected *Saccharina* samples. A preliminary report was presented at a symposium [16].

## Materials and methods

### Preparations and treatments of *Saccharina* samples

*Saccharina* samples were chosen from *Saccharina* specimens collected from the coasts off Hokkaido, Japan, from 1881 to 2008, which have been preserved at the Herbarium in the Hokkaido University Museum. The Herbarium is registered officially as the herbarium code (SAP) with the Index Herbariorum internationally. A total of 227 samples of *Saccharina* specimens (111 from the Japan Sea; 104 from the Pacific Ocean, and 12 from the Okhotsk Sea off Hokkaido) were analyzed. In addition, 27 other types of seaweed specimens collected from the Japan Sea off Hokkaido were further accessed at Hokkaido University Museum. When the samples were examined for this study, the specimens were still not fully collated and many of the specimens were not numbered. Currently this is being carried out. To compare between the present and previous nutrient conditions, fresh *Saccharina* samples from 2009 to 2014 were also collected from the same sites where the preserved *Saccharina* specimens had previously been collected. Fresh *Saccharina* is subject to aquatic harvest fishery rights and generally collecting kelp is prohibited. However, collection for the purpose of field studies is one of the exceptions, Hokkaido government especially issued specific permission for collecting the kelp. Furthermore, our field studies did not involve endangered or protected species. For analysis of *δ*^15^N, samples with few cracks, tears, and attached organisms on the surface were selected. After any dirt attached to the sample surface was carefully removed, samples were rinsed with filtered sea water, followed by washing with distilled water. In addition, three sea areas around Hokkaido, i.e., the Japan Sea, the Pacific Ocean, and the Okhotsk Sea were defined as follows respectively; from Soya to Cape Shiragami, Oshima, from Cape Shiragami to Cape Nossapu, Nemuro, and from Cape Nossapu to Abashiri (S1 Fig).

### Analyses of *δ*^15^N in *Saccharina*

The algal tissue of old and fresh *Saccharina* samples were oven-dried at 60°C and homogenized. Levels of *δ*^15^N in algal tissues were determined using an elemental analyzer equipped with an isotope ratio mass spectrometer (Fisons NA 1500-Finnigan MAT 252) at the Graduate School of Environmental Earth Science, Hokkaido University, Japan. The nitrogen isotope ratio was expressed as per mille (‰) deviation from the international standard (atmospheric air: AIR) as defined by the following equation: *δ*^15^N_Spl_ = (*R*_Spl_ / *R*AIR− 1), where *R* is ^15^N / ^14^N, Spl represents the sample. To calibrate raw *δ*^15^N values versus reference N_2_ gas to international standard, we used a two-point normalization method. The *δ*^15^Ns of working standards to calibrate the raw values were 13.8 and 6.0‰ using proline and tyrosine, respectively. At that time, *δ*^15^N value using a potassium nitrate (IAEA-NO-3) as international standard was 4.7‰. The analytical error was within ± 0.2‰.

## Results and discussion

**Fig 1:**
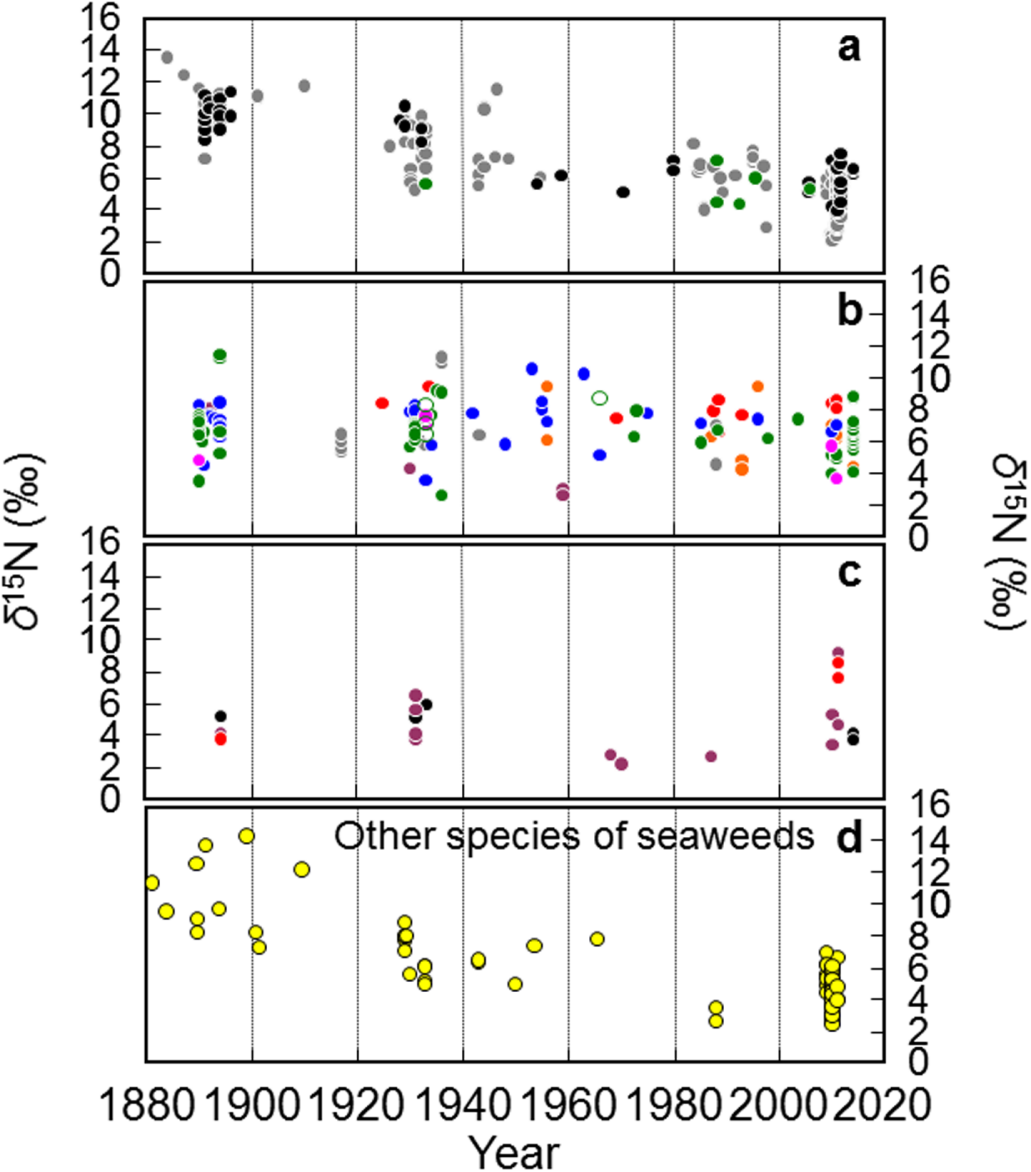
*δ*^15^N changes (1881–2014) in *Saccharina* specimens collected from coastal areas around Hokkaido, Japan. (a) The west coast of the Japan Sea off Hokkaido. (b) The coast of the Pacific Ocean off Hokkaido. (c) The coast of the Okhotsk Sea off Hokkaido. *Saccharina japonica* var. *ochotensis* designated with a black circle; *Saccharina japonica* var. *religiosa* designated with a gray circle; *Saccharina japonica* designated with a green solid circle; *Saccharina sculpera* designated with a pink circle; *Saccharina yendoana* designated with a green open circle; *Saccharina angustata* designated with a blue circle; *Saccharina coriacea* designated with a orange circle; *Saccharina longissima* designated with a red circle; *Saccharina japonica* var. *diabolica* designated with a purple circle. (d) Other species of seaweeds collected from the west coast of the Japan Sea off Hokkaido. The Herbarium in the Hokkaido University Museum is registered officially as SAP with the Index Herbariorum. SAP houses almost all marine benthic algal type specimens described by Japanese researchers. It is one of the marine benthic algal herbariums in the world (http://sap.museum.hokudai.ac.jp/index.html).

Fig 1 shows *δ*^15^N changes (1881–2014) in *Saccharina* specimens collected from coastal areas around Hokkaido, Japan: (a) The west coast of the Japan Sea off Hokkaido, (b) The coast of the Pacific Ocean off Hokkaido, (c) The coast of the Okhotsk Sea off Hokkaido. The *δ*^15^N of 100– to 135– year-old *Saccharina* samples collected from the west coast of the Japan Sea off Hokkaido (from 1881 to 1920) was significantly higher (mean ± S.D. 10.4 ± 1.1‰, n = 38) compared with that of freshly collected *Saccharina* (from 1971 to 2014) from the same region (5.3 ± 1.4‰, n = 105) in all *Saccharina* specimens: *Saccharina japonica* var. *ochotensis*, *Saccharina japonica* var. *religiosa*, and *Saccharina japonica*. Interestingly, the *δ*^15^N of other species of seaweeds collected from the west coast tended to be similar. In contrast, the *δ*^15^N of *Saccharina* collected from other coasts, i.e., the Pacific Ocean and Okhotsk Sea off Hokkaido, did not show the same trend as the west coast. The *δ*^15^N values of the three sea areas were divided into three periods (1881–1920, 1921–1970, and 1971–2014). By Kruskal-Wallis test, a significant difference was shown in the 9 groups (p < 0.01). Moreover, by Steel Dwass test, the *δ*^15^N of *Saccharina* samples collected from the west coast of the Japan Sea off Hokkaido (from 1881 to 1920) were significantly higher (p < 0.01) compared with values of other groups.

**Fig 2:**
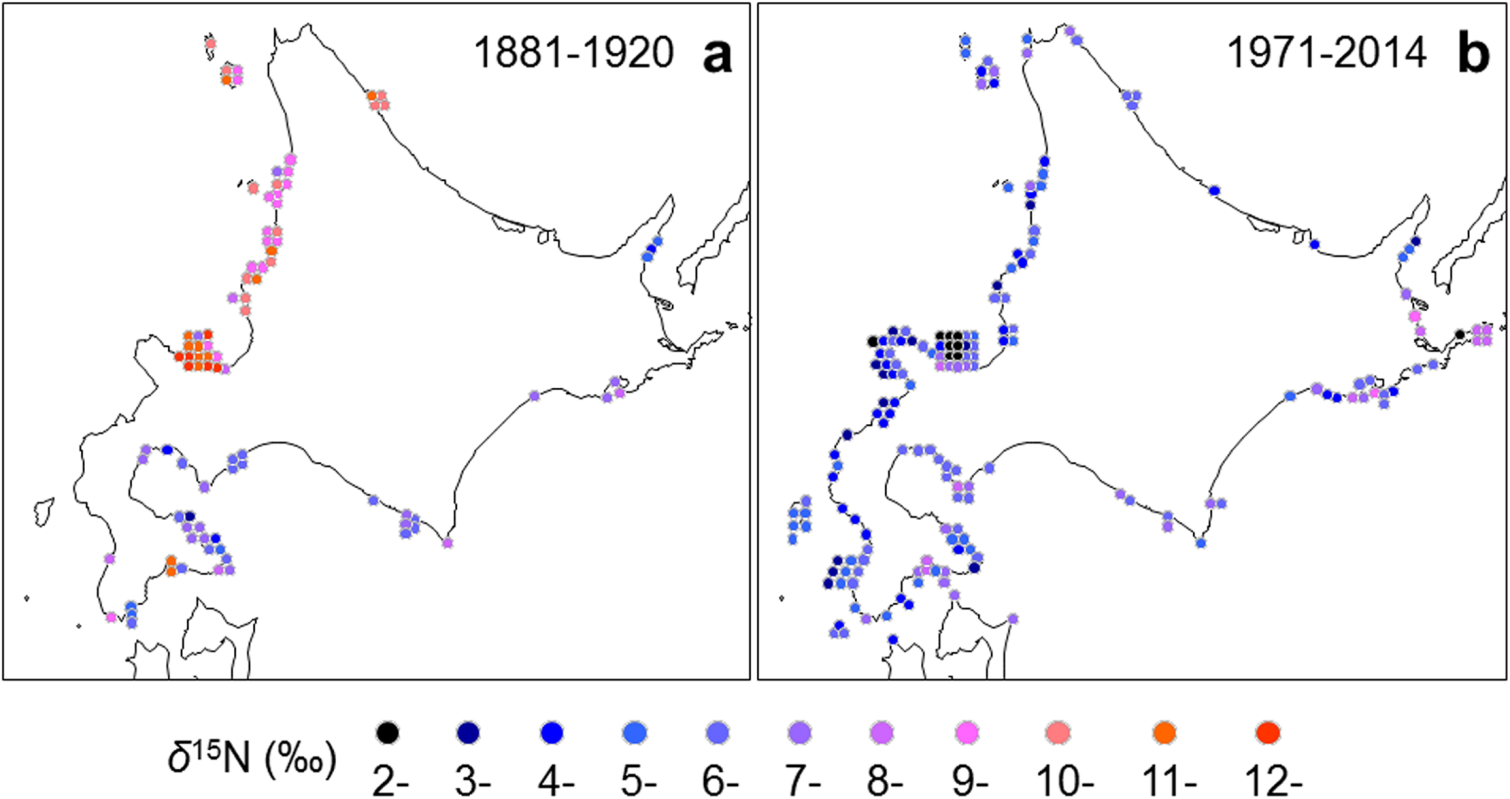
Maps of *δ*^15^N in the tissue of *Saccharina* collected from coastal areas around Hokkaido, Japan during the periods from 1881 to 1920 and 1971 to 2014. The *δ*^15^N ranges are classified by color differences.

The *δ*^15^N values of *Saccharina* and other species of seaweeds are plotted on a map of the coasts off Hokkaido divided into three periods (1881–1920, 1921–1970, and 1971–2014). From 1881 to 1920, *δ*^15^N showed high levels (10.4±1.5‰, n = 49) along the west coast of Hokkaido, and was higher than the *δ*^15^N (6.8 ± 1.6‰, n = 41) found along the other coasts of Hokkaido (Fig 2). In contrast, the *δ*^15^N was in the range of 2‰ to 9‰ along all coasts off Hokkaido from 1971 to 2014.

In our previous research, the *δ*^15^N-NO_3_ values which accounted for more than 90% of DIN from the west coast of the Japan Sea off Hokkaido were 5.3 ± 0.7‰ (n = 4), which was similar to the values from subarctic sea samples in modern times [5,17]. Furthermore, the *δ*^15^N values in freshly collected *Saccharina* from 2010 to 2014 were 5.0 ± 1.4‰ (n = 71). The *δ*^15^N values in *Saccharina* specimens collected from the west coast of the Japan Sea off Hokkaido from 1971 to 2009 showed 5.9 ± 1.3‰ (n = 34), which was similar to the *δ*^15^N values of NO_3_ and freshly collected *Saccharina*. These results indicate that the dominant source of DIN assimilated by *Saccharina* in this region was the Tsushima Warm Current water in recent times.

On the other hand, why were the *δ*^15^N values of 100– to 135– year-old *Saccharina* on the west coast of the Japan Sea off Hokkaido (from 1881 to 1920) significantly higher compared with those of other coasts and periods? When components in the tissues are used as environmental indicators, preservation methods for specimens have to be considered. Over periods of multiple decades, in organic matter such as in seaweeds, the lighter ^14^N of the nitrogen components will have been selectively removed compared with the heavier ^15^N. The different reactivity between ^14^N and ^15^N because of the mass difference, results in an elevation of the level of *δ*^15^N [18,19]. *Saccharina* specimens have been preserved in museums for up to 135 years. However, all specimens have been preserved under the same desiccated conditions to minimize decomposition. The *δ*^15^N of older specimens may be affected by decomposition and would be expected to increase with similar trends for specimens from all coasts off Hokkaido. However, only *δ*^15^N values in *Saccharina* on the west coast of the Japan Sea off Hokkaido from 1881 to 1920 were higher. Therefore, potential effects of decomposition are concluded not to account for the high *δ*^15^N values observed in older specimens.

Furthermore, on biological assimilation, the isotopically lighter ^14^N is selectively used, resulting in lower *δ*^15^N values in algal tissue than the *δ*^15^N of NO_3_ (isotopic fractionation on biological assimilation). The *δ*^15^N in the tissue elevates to close to the *δ*^15^N of NO_3_ using high *δ*^15^N of the remaining NO_3_ according to the Rayleigh distillation model. When NO_3_ is completely depleted, the *δ*^15^N values in the tissue of primary producers are finally equal to the *δ*^15^N of NO_3_. Moreover, as mentioned above, the *δ*^15^N-NO_3_ values from the west coast of the Japan Sea off Hokkaido were 5.3 ± 0.7‰, as such *δ*^15^N values of algal tissues will not increase to 10‰ by isotopic fractionation on biological assimilation. Therefore, it was considered that the high *δ*^15^N reflected the in situ water column condition, and provides integrated information on the biologically available form in the marine ecosystem. Several factors may account for the high *δ*^15^N observed in the tissue of *Saccharina* collected from the west coast of the Japan Sea off Hokkaido from 1881 to 1920, including the following: denitrification; and difference of nitrogen sources. These possibilities are discussed below.

### Denitrification

In a reductive environment, denitrifying bacteria use NO_3_-O as a source of oxygen releasing N_2_ gas, namely denitrification. In this reaction, isotopically lighter ^14^NO_3_ is preferentially used, and heavier ^15^NO_3_ remains relatively more, leading to a higher *δ*^15^N value of NO_3_. With assimilation of the remaining NO_3_-N, the *δ*^15^N of primary producers could be expected to increase further than 10‰ [20]. For example, three sea areas are major denitrification regions, i.e., the Arabian Sea, the east tropical North Pacific Ocean, and the South Pacific Ocean, which are upwelling and tropical areas, are characterized by high productivity and strong stratification. Decomposition of a lot of sinking organic matter from the surface layer is dominant in the intermediate layer where minimal exchange of seawater occurs. This property promotes the progress of an O_2_ depleted reductive environment, and results in increasing *δ*^15^N-NO_3_ by denitrification. With assimilation of this NO_3_-N, the *δ*^15^N in *Saccharina* would also be expected to increase. However, as the west coast of the Japan Sea off Hokkaido is characterized by vertical mixing and high biological diversity, it was unlikely that hypoxic conditions causing denitrification have widely occurred. Furthermore, the water mass of the Japan Sea is generally classified as “Tsushima Warm Current Water” distributed from the surface layer (to approximately <300m depth) which flows north along the coast Japan from the Tsushima Straits, and the “Japan Sea Proper Water” distributed in the middle and deeper layers. The origin of the Japan Sea Proper Water is different from the Tsushima Warm Current Water, and is formed by the subduction of oxygen-rich surface seawater cooling on the northwestern part of the Japan Sea [21,22]. Therefore, dissolved oxygen concentration is 230–260 μmol kg^-1^ and oxygen saturation is more than 60% of the surface sea layer, which is higher than concentrations of other seas. Oba and others reported that the Japan Sea has been an oxidative environment since at least 6,300 years ago [23]. Therefore, the hypothesis that pelagic nitrate partly denitrified in the middle or deeper layer and subsequently inflowed to the west coast of Hokkaido during the 134 year period (1881–2014) is concluded not to account for the high *δ*^15^N observed in *Saccharina* specimens.

### Difference of nitrogen sources

#### Anthropogenic nitrogen

All consumers, zooplankton, fish and birds show stepwise enrichment of ^15^N with the increasing trophic level [24,25]. Humans which form a high trophic level organism in food chains also shows high levels of *δ*^15^N in body components. Moreover, NH_3_ volatilization selectively removes isotopically lighter ^14^N to the atmosphere, with heavier ^15^N remaining at relatively higher levels. These properties promote high *δ*^15^N of anthropogenic DIN in human activities. The *δ*^15^N of DIN in coastal areas may be affected by input of anthropogenic DIN and would be expected to increase [26,27]. With assimilation of anthropogenic DIN, the *δ*^15^N in *Saccharina* would be also expected to increase. Previous studies reported that the population density, type of land-use, which are often used as indicators of anthropogenic nitrogen impact, are correlated with *δ*^15^N levels in organisms from watersheds [9,28–30]. For example, it was reported that there was a positive correlation of the *δ*^15^N of primary consumers and the human population density in each watershed worldwide [31]. The history of wastewater treatment in Hokkaido, Japan began about 120 years ago with the development of Hokkaido (under directions of the Meiji government, 1868–1912), and treated agricultural, industrial, and domestic wastewater. Agricultural wastewater was mainly discharged along the coasts of the Japan Sea and the Pacific Ocean since the 1880s. Industrial wastewater was mainly discharged from the coal industry which was developed on the coast of the Japan Sea and the Pacific Ocean from 1950 to 1960. Moreover, the population of Ishikari has increased since the 1880s, and the discharge of domestic wastewater has increased along the coast of the Japan Sea. In this study, the relationship between the population density of the watershed in which *Saccharina* specimens were collected and the *δ*^15^N levels in *Saccharina* was examined. The *δ*^15^N levels did not show the same trend as population density. For example, on the west coast of the Japan Sea off Hokkaido, the *δ*^15^N levels in *Saccharina* collected from 1881 to 1920 were higher than the levels collected from 1971 to 2014, although the population density from 1881 to 1920 was lower.

#### Nitrogen derived from organisms

Organisms moving between ecosystems transport and translocate essential resources such as organic matter, nutrients, and minerals as vectors in the form of carcasses and excrement. Previous studies examining the contributions of anadromous fishes (spawning salmon [32,33], herring [34–37]), seabirds [38,39], sea turtles [40], and insects [41] have suggested that the uptake of essential substances released by decomposition is an important way of enhancing the biological productivity of ecosystems. Excrement, parental bodies of spawning salmon that become deposited in terrestrial environments are substances synthesized in the sea. Moreover, seabirds spend most of their life on the sea eating marine foods. They transport nutrients and organic matter to the land from the sea. These high trophic level organisms in food chains also show high levels of *δ*^15^N in their components. Furthermore, NH_3_ volatilization selectively removes isotopically lighter ^14^N to the atmosphere, with heavier ^15^N remaining relatively more. These properties promote *δ*^15^N enrichment of DIN derived from organisms. The *δ*^15^N of DIN in coastal areas may be affected by the input of DIN derived from organisms and would be expected to increase. With assimilation of DIN, the *δ*^15^N in *Saccharina* would also be expected to increase.

**Fig 3:**
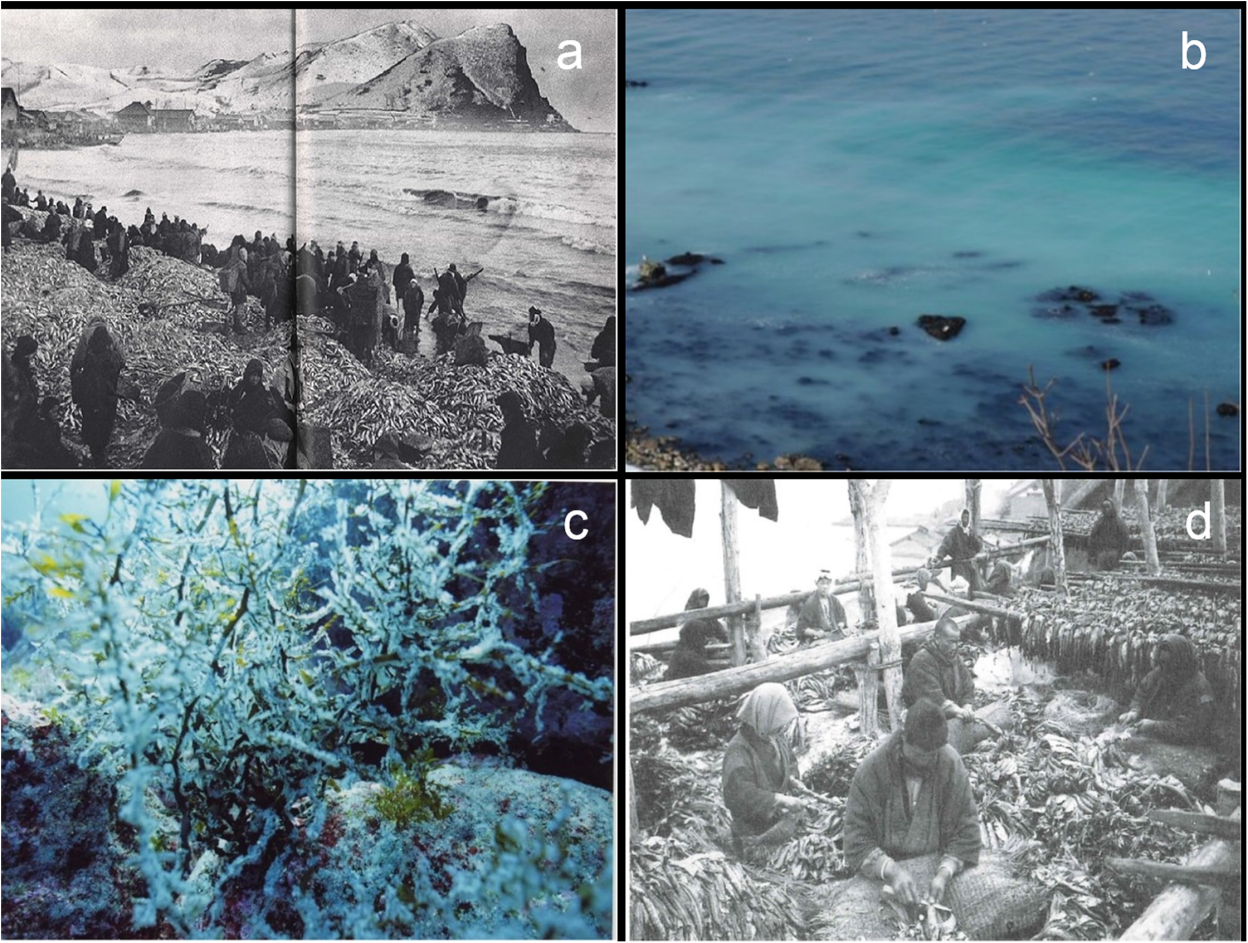
Historic photographs from the west coast of the Japan Sea off Hokkaido, Japan. (a) A large number of spawning herring that became stranded on the shore (in Yoichi, 1919), reprinted from Mitsuru Hayashi under a CC BY license, with permission from Mitsuru Hayashi, original copyright 1919. (b) Phenomenon of milky-white turbidity of the sea surface due to herring spawning, called “*kuki*”, in coastal areas (in Otaru, 2011) reprinted from Takanori Kuribayashi under a CC BY license, with permission from Takanori Kuribayashi, original copyright 2011. (c) Seaweed bed covered with herring eggs (off Haboro, 2004), reprinted from Shoichi Akaike under a CC BY license, with permission from Shoichi Akaike, original copyright 2004. (d) Processing herring for use in “*migaki-nishin*,” a dried and sliced herring food (in Suttsu, 1911 or 1914), reprinted from *Suttsu gojyuwa* under a CC BY license, with permission from Tatsuya Yamamoto, original copyright 2014.

Based on these observations, one hypothesis has been suggested that nutrients transported by spawning herring may explain the previous nutrient conditions along the west coast off Hokkaido (Fig 3A). From 1880 to 1920, most of the Hokkaido fisheries had been for spawning herring (Pacific herring; *Clupea pallasii*) on the west coast of the Japan Sea off Hokkaido, representing more than 90% of herring spawning around all coasts off Hokkaido (S2 Fig). An average of approximately 600,000 tons of spawning herring were caught in this region between 1880 and 1920; this is 500–1000 times the number of spawning herring caught in recent years [4].

Spawning herring produce sperm and eggs in coastal areas. Male herring spawning leads to an appearance of milky-white turbidity at the sea surface, called “*Kuki*” in this region (Fig 3B). Algal beds are covered with eggs spawned by female herring, and are characterized by the presence of a demersal, sticky substance over the seaweeds (Fig 3C). From 1880 to 1920, a large number of spawning herring annually migrated to the algal beds with a substantial spawning event and subsequent large-scale “*Kuki*”. A previous report suggested that the reduction of herring spawning may have resulted in decreased yields and biomass of *Saccharina* based on multiple correlations among *Saccharina* yields, herring catches, and the coastal temperatures along the southwest coast off Hokkaido [42]. Hay and Fulton, proposed that herring spawning products might be a source of nutrients to promote secondary production in the Strait of Georgia [43].

Furthermore, in the late 19^th^ century approximately 85% of the fertilizers produced in Japan were made from herring [44]. Although the use of herring as a fertilizer decreased in the 1900s, the use of “*Migaki-Nishin*,” a dried and sliced herring food, and “*Kazunoko*,” herring roe, increased in the 1920s. These products were processed along the coast (Fig 3D) and the abundance of herring processing residue results in a large amount of nitrogen input [45]. Additionally, nitrogen components in fish processing residues are remineralized after being added to seawater. In fact, some fishermen have recognized that large-scale *Saccharina* beds had formed when the herring residue had been inputted in coastal waters, there are some working projects to use fish processing residues as fertilizer in coastal sea areas. It is impossible to directly show the high *δ*^15^N of DIN in the Japan Sea before 1920 because of the lack of previous seawater samples and difficulty of the approach using sediment cores. However, this hypothesis may be consistent with the deposition of a large quantity of herring residue-derived DIN along the west coast of the Japan Sea off Hokkaido. We found that the *δ*^15^N values of herring eggs, sperm, and herring processing residue were high: 10.8 ± 0.4, 9.1 ± 0.3, and 11.8 ± 1.0 (n = 10), respectively (S2 Table). As herring have not been caught in recent years to the same extent as the period 1880 to 1920, the same environmental conditions as in the past cannot be completely reconstructed. Therefore, in a preliminary laboratory experiment, filtrated seawater including herring eggs, sperm, and residue in a container was analyzed. As a result, it was found that the DIN concentration increased (S3 Fig). Moreover, when male herring spawning causes the small scale appearance of milky-white turbidity at the sea surface, called “*Kuki*” in the west coast off Hokkaido in recent years, seawater was analyzed. As a result, the PON (particulate organic nitrogen) concentration, PON-*δ*^15^N value, DIN concentration, and *δ*^15^N value in algal tissue of seaweeds were found to increase (S3 Table). For this mechanism to work, the decomposition of herring eggs and sperm, or residue would have to be transformed into available DIN for assimilation by *Saccharina*. With complete assimilation the *δ*^15^N of primary producers could be expected to increase further than 10‰. Therefore, it is possible that the DIN assimilated by *Saccharina* is derived from herring eggs, sperm or residue which were distributed along the west coast of the Japan Sea off Hokkaido 100–135 years ago.

Seasonal changes of nutrient concentrations on the west coast of the Japan Sea off Hokkaido, an area strongly influenced by the Tsushima Warm Current water, have been observed by monitoring the nutrient status of the sea current [46,47]. DIN concentrations reach a maximum level of about 5 μM in winter, significantly decrease in spring, and reach a minimum of almost 0 μM from spring to autumn. During such cycles, spring is the *Saccharina* growth season although there is a decrease in DIN concentrations. Interestingly, previous stocks of herring also genetically differed from the present stocks [48]. The present stocks of herring, called the “*Ishikari Bay group*” spawn in winter, and spend their lifetime in Ishikari Bay off Hokkaido. On the other hand, previous stocks of herring called the “*Hokkaido and Sakhalin group*,” spawned in spring (for three months from March to May) [4,44]. The juveniles that hatched along the west coast off Hokkaido migrated to the east coast of the Okhotsk Sea off Hokkaido and the Pacific Ocean off Iwate through the Soya strait. They returned to spawn in this region again in spring which is the *Saccharina* growth season although the DIN concentrations in the Tsushima Warm Current water decrease in spring. Calculating the herring catches, it was estimated that approximately 1 ton of spawning herring were caught every day in the spring between 1880 and 1920 on the west coast of the Japan Sea off Hokkaido. This suggests that a large amount of herring-derived nutrients from feeding in the Okhotsk Sea and the Pacific Ocean could have become available along the west coast of the Japan Sea off Hokkaido which was characterized by oligotrophy. In the museum, there are many specimens collected from spring to autumn. The *δ*^15^N value in the spring specimens tended to be similar to the specimens in the other seasons. The season of nutrient supply via DIN from the Tsushima Warm Current water is typically only winter around coasts off Hokkaido, the *δ*^15^N in algal tissue integrally includes *δ*^15^N of DIN assimilated in winter. However, historically spring was also a herring-derived nutrient supply season on the west coast off Hokkaido, the *δ*^15^N in algal tissue integrally include *δ*^15^N of DIN derived from spawning herrings.

Furthermore, nutrients in seawater often limit the kelp production as a “Bottom-up effect” [49]. Nutrient supply is essential for growth enhancement of kelp [1,45,50,51]. Our previous experiments suggested that *Saccharina* growth was promoted by fertilization with DIN (S4 Fig). Nutrient supply was examined by adding (NH_4_)_2_SO_4_ fertilizer which shows lower *δ*^15^N than *δ*^15^N-NO_3_ of natural seawater on the southwest coast of Hokkaido, Japan [52]. The *δ*^15^N values in seaweeds before fertilization were close to *δ*^15^N-NO_3_ in the Tsushima Warm Current. After fertilization, *Saccharina japonica* var. *religiosa* growth promotion and algal biomass enhancement were observed at the fertilization point compared with the natural site (S4 Fig). The *δ*^15^N values in algal tissue were depleted at the fertilization point, and approached levels before fertilization with distance from the fertilization point (S5 Fig). These results indicate that the nutrient supply was reflected in the *δ*^15^N values of algal tissue. Moreover, on the west coast of Hokkaido, Japan, the *δ*^15^N of algal tissue was increased further than 10‰ by fertilizing fish processing residue in spring (S4 Table). The fertilizer was mainly made from herring, we confirmed that the mineralization way and rate of the fertilizer were similar to those of herring. The periods when the fertilizer N in the experiment of S4 Table added was appropriate for the model of the past herring events. The fertilization site was appropriate as a representative place of the west coast of Hokkaido. In our preliminary laboratory experiment, one block (30×30×30 cm) mainly made from herring was fertilized in a panlite tank and a rate of 200L day^-1^ of seawater was injected there under flowing. As a result, the DIN concentration was primarily high and the *δ*^15^N in algal tissue of *Saccharina japonica* var. *religiosa* increased with time (S6 Fig).

**Fig 4:**
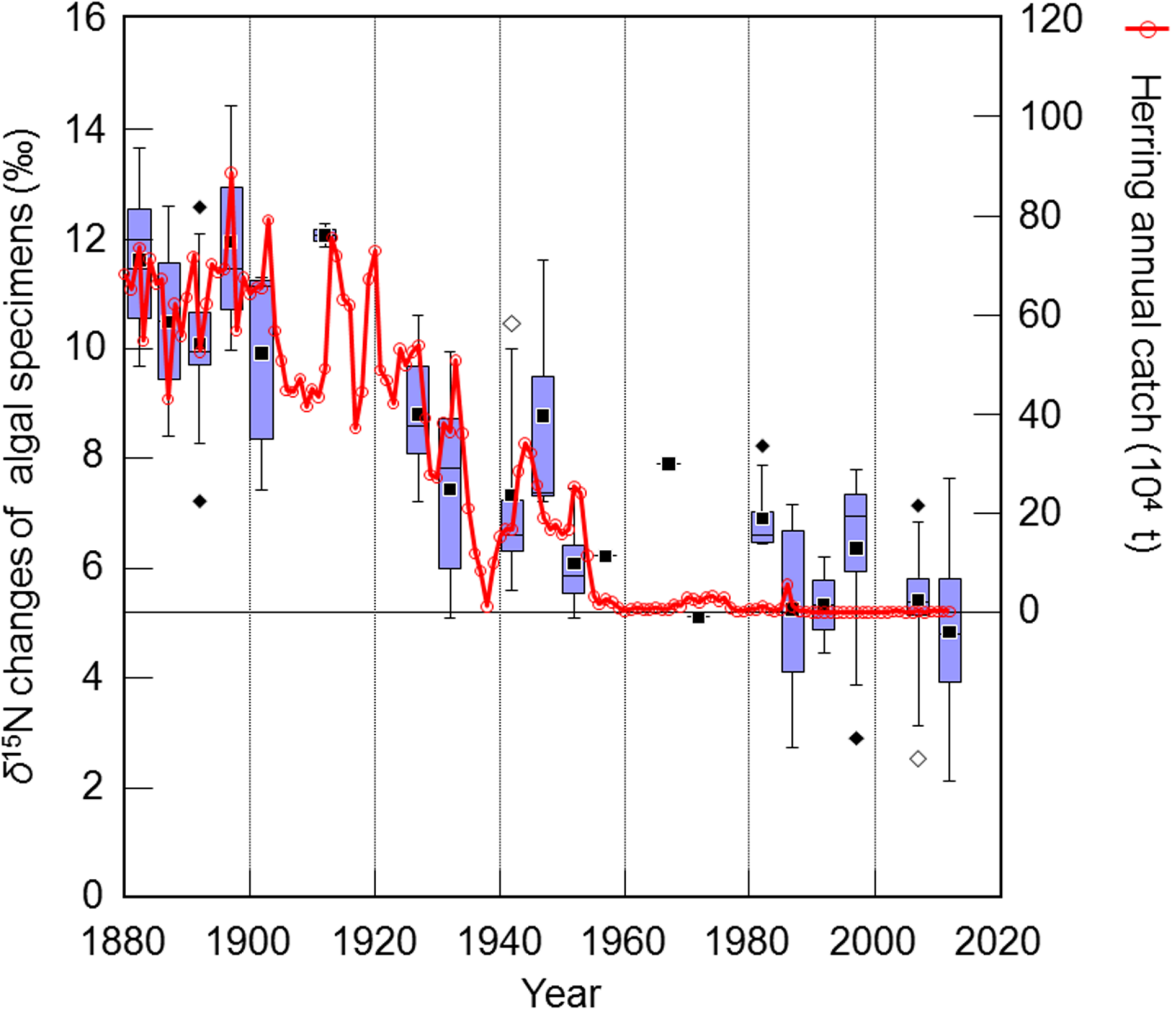
Comparison of changes in *δ*^15^N values in the tissues of *Saccharina* specimens and herring catches along the west coast of the Japan Sea off Hokkaido, Japan [4,53–55]. The changes in *δ*^15^N in algal tissue are shown by box-and-whisker plots for each 5 years from 1881 to 2014. The bottoms and tops of boxes indicate 25% and 75% uncertainty ranges, respectively, and the line in the box indicates the median. Upper and lower error bars indicate the largest value below the upper inner fence and the smallest value above lower inner fence, respectively. These values were 75% + 1.5 × (75%–25%) and 25%–1.5 × (75%–25%), respectively, indicated by closed diamonds. The upper and lower outer fences were 75% + 3 × (75%–25%) and 25%–3 × (75%–25%), respectively, indicated by open diamonds.

In this study, comparison between *δ*^15^N of algal tissue in *Saccharina* and herring catches on the west coast of the Japan Sea off Hokkaido were carried out. The *δ*^15^N of historical changes correlated with herring catches (r = 0.80, p < 0.01, n = 256) (Fig 4). Considering these results, it is plausible that the DIN assimilated by *Saccharina* was derived from spawning herrings (eggs, sperm, or processing residues) acting as organisms forming a vector for nutrients from their feeding grounds (Okhotsk Sea and Pacific Ocean) to their spawning area (west coast of Hokkaido, Japan Sea) 100–135 years ago. Our findings support the hypothesis of so-called “herring-derived nutrients”.

## Supporting information

S1 Fig The locations of the sea off the coast of Hokkaido, Japan. Three coastal areas, the Japan Sea, the Pacific Ocean, and the Okhotsk Sea around Hokkaido are defined as follows respectively; from Soya to Cape Shiragami, Oshima, from Cape Shiragami to Cape Nossapu, Nemuro, and from Cape Nossapu to Abashiri.
(PDF)

S2 Fig Annual changes in the main catches in Hokkaido, Japan from 1880 to 2013. The bold red line between 1880 and 1886 represented annual changes of herring catches around all coasts off Hokkaido. From 1880 to 1920, most Hokkaido fisheries had been herring on the west coast of the Japan Sea off Hokkaido, representing more than 90% of herring spawning around all coasts off Hokkaido [4]. An average of approximately 600,000 tons of spawning herring were caught in this region between 1880 and 1920; this is between 500−1000 times the number of spawning herring caught in recent years.
(PDF)

S3 Fig DIN variations of herring sperm, eggs, and residue in relation to incubation periods. In incubation experiments, 1 g of herring sperm and eggs were respectively added to 1 L of seawater adjusted to 15°C in the bottle under non-flowing condition. Furthermore, 1 L of herring residue was adjusted to 15°C in the bottle under non-flowing condition. Each DIN concentration was determined for aerated bottles.
(PDF)

S4 Fig **Comparison of growth of *Saccharina japonica* var. *religiosa* at the fertilization point (a) with the non-fertilization point (b).** This photograph was provided by the Fisheries Research Department, Hokkaido Research Organization, Japan.
(PDF)

S5 Fig *δ*^15^N changes in algal tissue in relation to distance from the fertilizing point. Nutrient supply was examined by adding (NH_4_)_2_SO_4_ (-4.3 ± 2.0 ‰, n = 10) fertilizer which shows lower *δ*^15^N than *δ*^15^N-NO_3_ of natural seawater on the southwest coast of Hokkaido, Japan.
(PDF)

S6 Fig DIN variations and *δ*^15^N changes in *Saccharina japonica* var. *religiosa* in fertilization and non-fertilization in relation to incubation periods. In incubation experiments, one block (30×30×30 cm) was fertilized in a panlite tank and seawater adjusted to 15°C added at a rate of 200 L day^-1^. DIN concentrations in sea water using a panlite tank and *δ*^15^N changes in *Saccharina japonica* var. *religiosa* were determined.
(PDF)

S7 Fig The granted permission from the copyright holder to publish Fig 3A under a CC BY license. (PDF)

S8 Fig The granted permission from the copyright holder to publish Fig 3C under a CC BY license. (PDF)

S9 Fig The granted permission from the copyright holder to publish Fig 3D under a CC BY license. (PDF)

S1 Table *δ*^15^N values (1881–2014) in *Saccharina* and other species of seaweeds specimens collected from coastal areas around Hokkaido, Japan. (PDF)

S2 Table *δ*^15^N values of herring sperm, eggs and herring processing residue. (PDF)

S3 Table DIN and PON concentration, *δ*^15^N-PON values, and *δ*^15^N in seaweeds on the coast off Otaru (Shukutsu) and control site (Yoichi) on 8 February 2013, Otaru (Asari) and control site (Yoichi) on 14 February 2013 when male herring spawning caused the appearance of milky-white turbidity at the sea surface, called “*Kuki*” though it was a small-scale event compared with the past. *δ*^15^N in seaweeds on the coast off Otaru (Shukutsu and Asari) were high in 9 March 2013 after “*Kuki*”.
(PDF)

S4 Table *δ*^15^N values in algal tissues of *Saccharina japonica* var. *religiosa* before and after fertilization at the fertilization point, non-fertilization point, and the fertilizer made from fish processing residue with wood chip (“Fertilizer”). (PDF)
