# Supplementary material for: Historical δ15N records of Saccharina specimens from oligotrophic waters of Japan Sea (Hokkaido)
Source: PLoS One. 2017 Jul 12;12(7):e0180760. doi: 10.1371/journal.pone.0180760 (PMC5507519; doi:10.1371/journal.pone.0180760)
Supplement: S1 Fig — Three coastal areas, the Japan Sea, the Pacific Ocean, and the Okhotsk Sea around Hokkaido are defined as follows respectively; from Soya to Cape Shiragami, Oshima, from Cape Shiragami to Cape Nossapu, Nemuro, and from Cape Nossapu to Abashiri. (PDF) [file pone.0180760.s001.pdf]

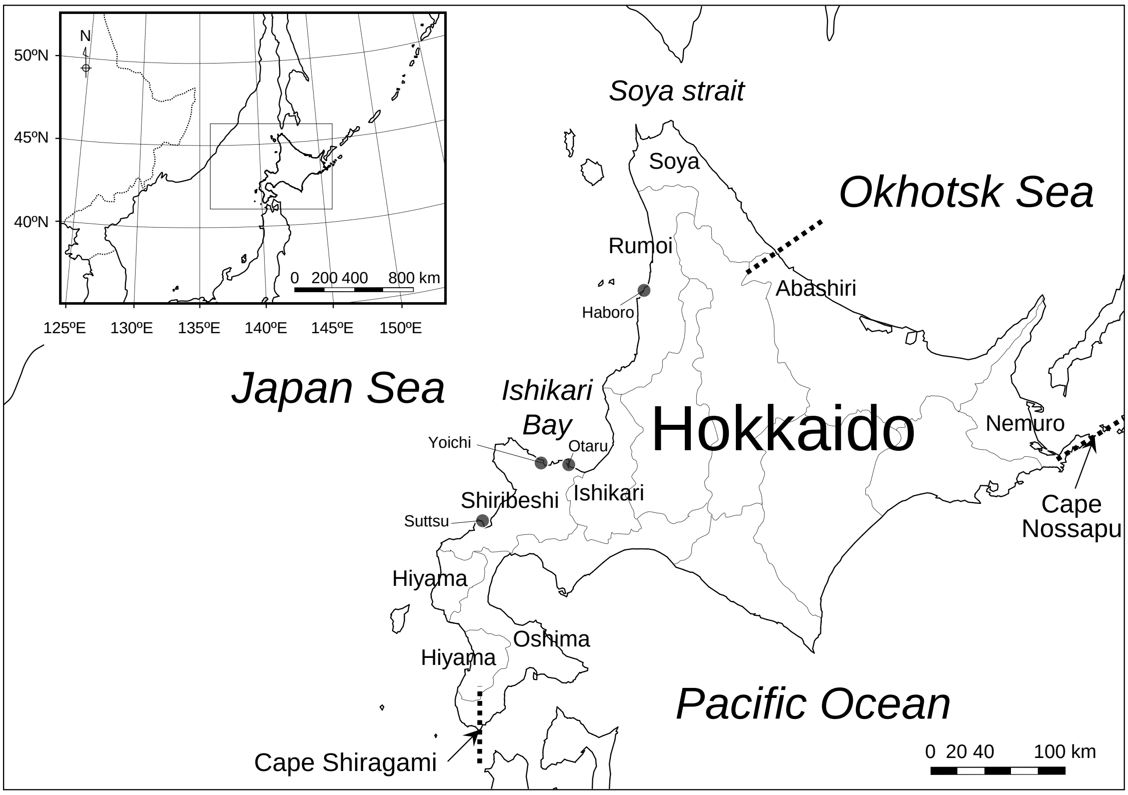

S1 Fig. The locations of the sea off the coast of Hokkaido, Japan. Three coastal areas, the Japan Sea, the Pacific Ocean, and the Okhotsk Sea around Hokkaido are defined as follows respectively; from Soya to Cape Shiragami, Oshima, from Cape Shiragami to Cape Nossapu, Nemuro, and from Cape Nossapu to Abashiri.
