# Supplementary material for: Historical δ15N records of Saccharina specimens from oligotrophic waters of Japan Sea (Hokkaido)
Source: PLoS One. 2017 Jul 12;12(7):e0180760. doi: 10.1371/journal.pone.0180760 (PMC5507519; doi:10.1371/journal.pone.0180760)
Supplement: S2 Fig — The bold red line between 1880 and 1886 represented annual changes of herring catches around all coasts off Hokkaido. From 1880 to 1920, most Hokkaido fisheries had been herring on the west coast of the Japan Sea off Hokkaido, representing more than 90% of herring spawning around all coasts off Hokkaido [4]. An average of approximately 600,000 tons of spawning herring were caught in this region between 1880 and 1920; this is between 500−1000 times the number of spawning herring caught in recent years. (PDF) [file pone.0180760.s002.pdf]

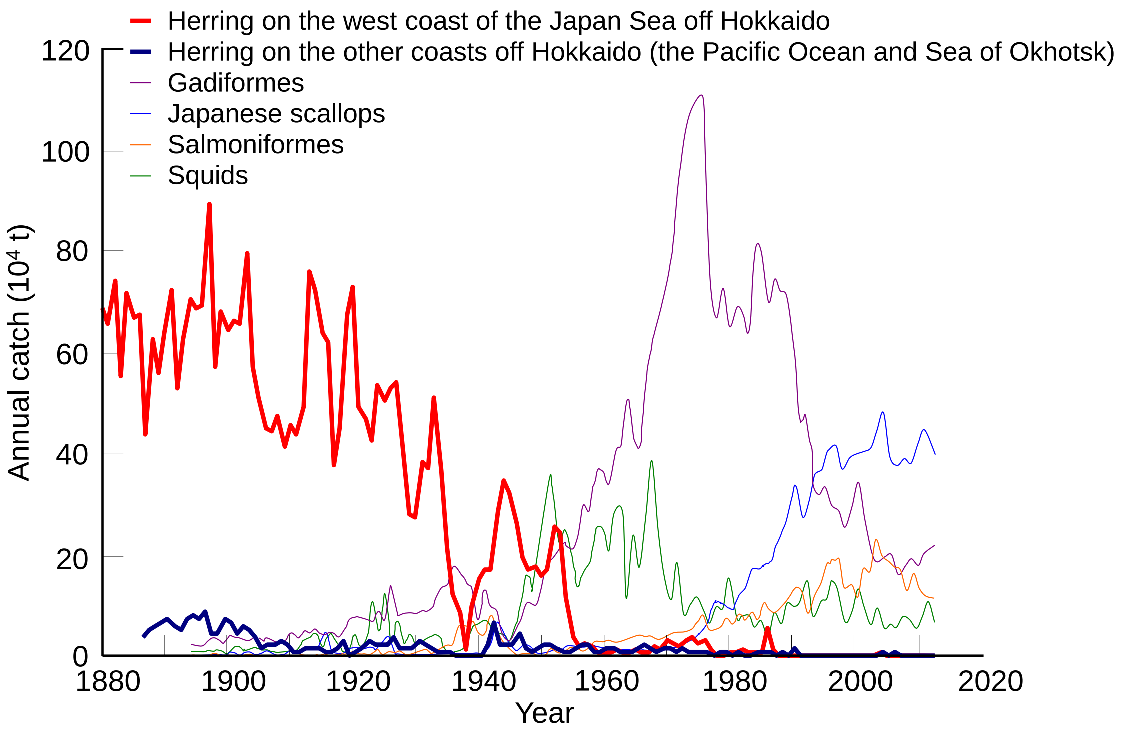

S2 Fig. Annual changes in the main catches in Hokkaido, Japan from 1880 to 2013. The bold red line between 1880 and 1886 represented annual changes of herring catches around all coasts off Hokkaido. From 1880 to 1920, most Hokkaido fisheries had been herring on the west coast of the Japan Sea off Hokkaido, representing more than 90% of herring spawning around all coasts off Hokkaido [4]. An average of approximately 600,000 tons of spawning herring were caught in this region between 1880 and 1920; this is between 500−1000 times the number of spawning herring caught in recent years.
