# Supplementary material for: Historical δ15N records of Saccharina specimens from oligotrophic waters of Japan Sea (Hokkaido)
Source: PLoS One. 2017 Jul 12;12(7):e0180760. doi: 10.1371/journal.pone.0180760 (PMC5507519; doi:10.1371/journal.pone.0180760)
Supplement: S3 Fig — In incubation experiments, 1 g of herring sperm and eggs were respectively added to 1 L of seawater adjusted to 15°C in the bottle under non-flowing condition. Furthermore, 1 L of herring residue was adjusted to 15°C in the bottle under non-flowing condition. Each DIN concentration was determined for aerated bottles. (PDF) [file pone.0180760.s003.pdf]

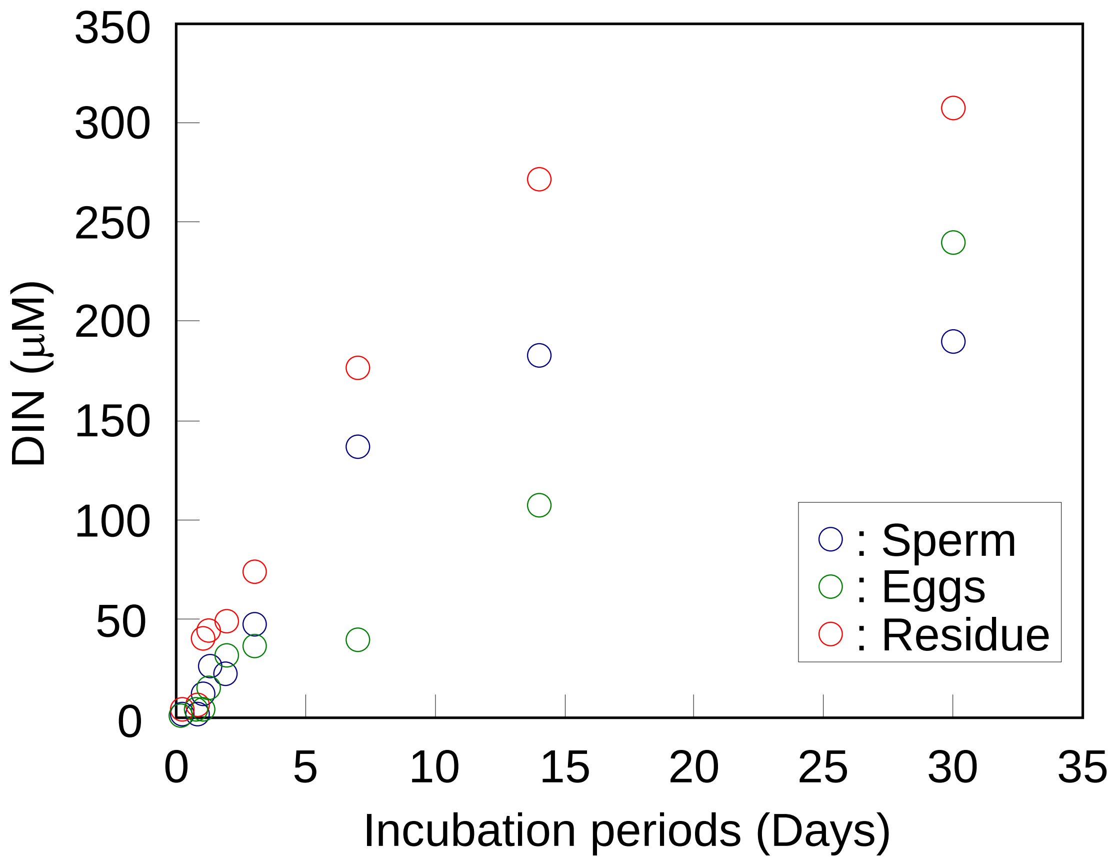

S3 Fig. DIN variations of herring sperm, eggs, and residue in relation to incubation periods. In incubation experiments, 1 g of herring sperm and eggs were respectively added to 1 L of seawater adjusted to 15 ^{o}C in the bottle under non-flowing condition. Furthermore, 1 L of herring residue was adjusted to 15 ^{o}C in the bottle under non-flowing condition. Each DIN concentration was determined for aerated bottles.
