# Supplementary material for: Historical δ15N records of Saccharina specimens from oligotrophic waters of Japan Sea (Hokkaido)
Source: PLoS One. 2017 Jul 12;12(7):e0180760. doi: 10.1371/journal.pone.0180760 (PMC5507519; doi:10.1371/journal.pone.0180760)
Supplement: S4 Fig — Comparison of growth of Saccharina japonica var. religiosa at the fertilization point (a) with the non-fertilization point (b). This photograph was provided by the Fisheries Research Department, Hokkaido Research Organization, Japan. (PDF) [file pone.0180760.s004.pdf]

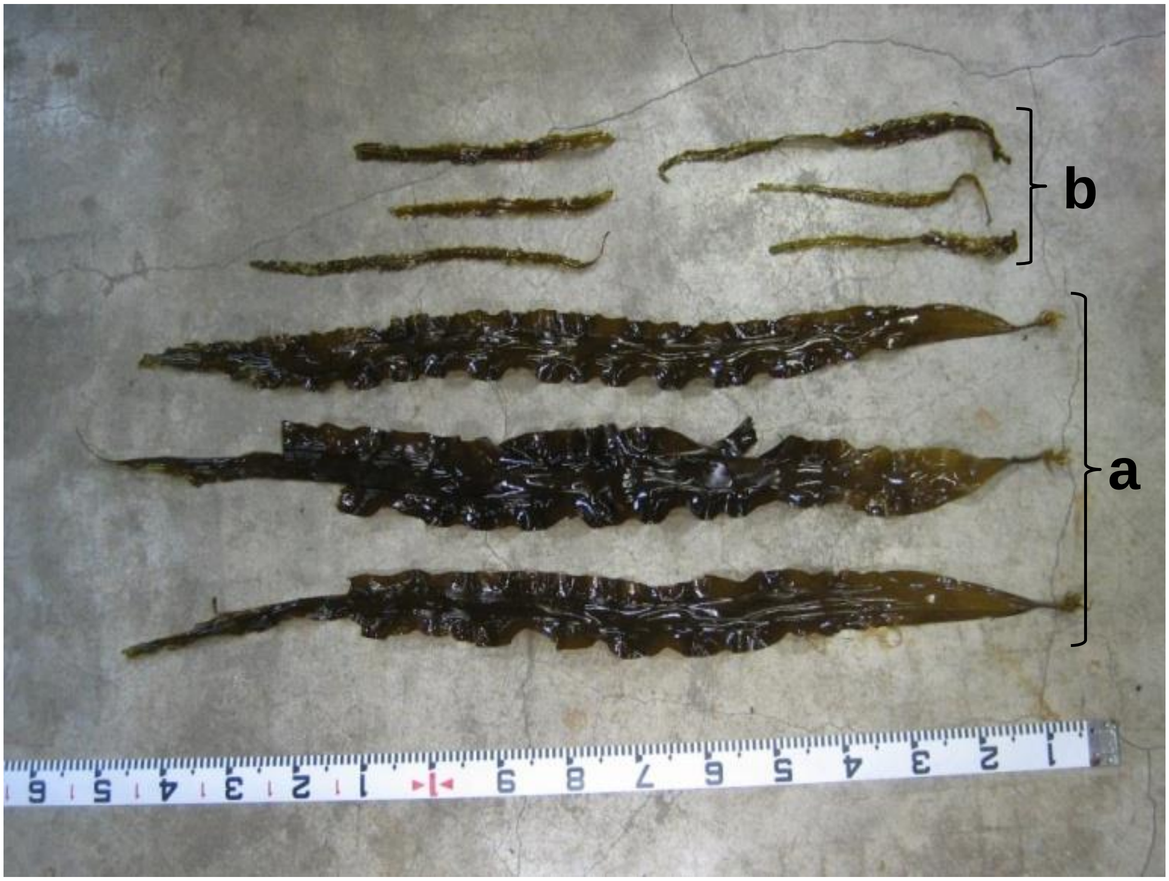

S4 Fig. Comparison of growth of *Saccharina japonica* var. *religiosa* at the fertilization point (a) with the non-fertilization point (b). This photograph was provided by the Fisheries Research Department, Hokkaido Research Organization, Japan.
