# Supplementary material for: Historical δ15N records of Saccharina specimens from oligotrophic waters of Japan Sea (Hokkaido)
Source: PLoS One. 2017 Jul 12;12(7):e0180760. doi: 10.1371/journal.pone.0180760 (PMC5507519; doi:10.1371/journal.pone.0180760)
Supplement: S5 Fig — Nutrient supply was examined by adding (NH4)2SO4 (-4.3 ± 2.0 ‰, n = 10) fertilizer which shows lower δ15N than δ15N-NO3 of natural seawater on the southwest coast of Hokkaido, Japan. (PDF) [file pone.0180760.s005.pdf]

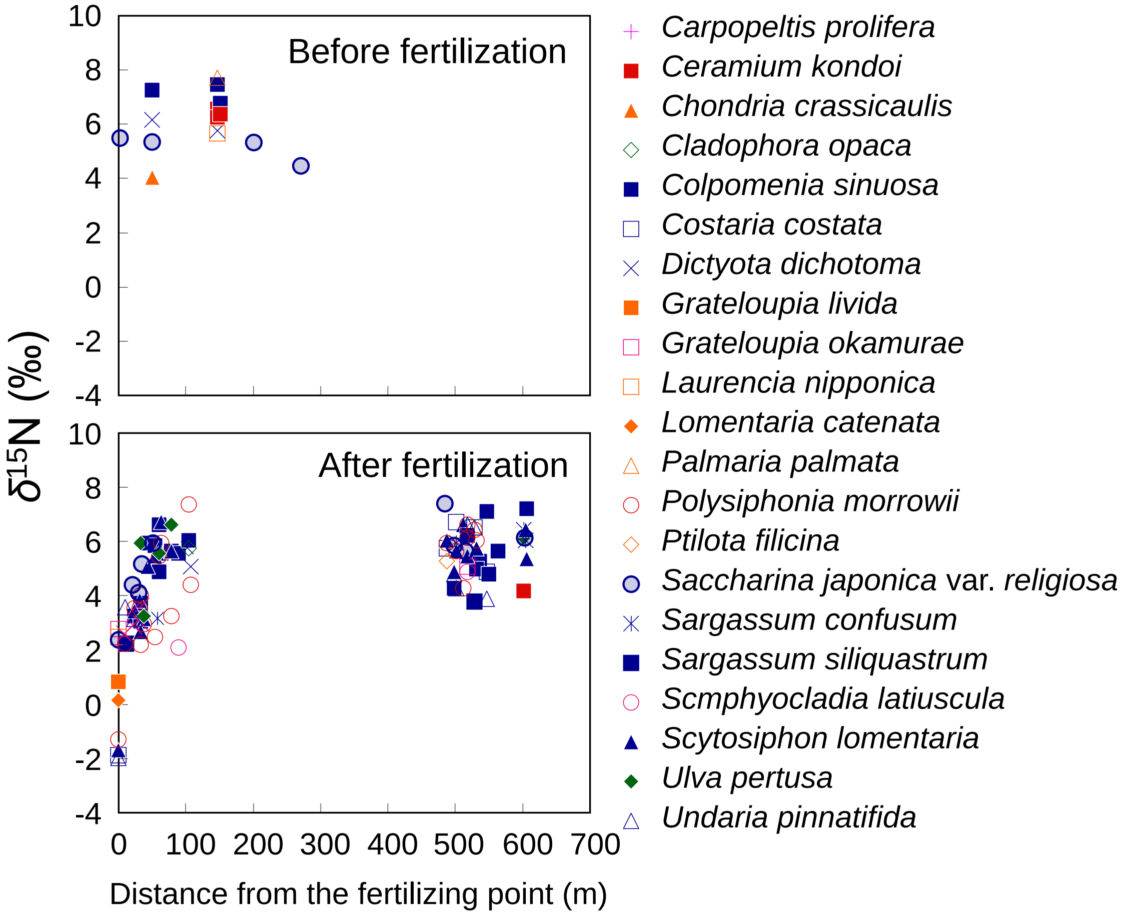

S5 Fig. δ^{15}N changes in algal tissue in relation to distance from the fertilizing point. Nutrient supply was examined by adding $(NH_4)_2SO_4$ (-4.3 ± 2.0 ‰, n= 10) fertilizer which shows lower δ^{15}N than δ^{15}N-NO_3 of natural seawater on the southwest coast of Hokkaido, Japan.
