# Supplementary material for: Historical δ15N records of Saccharina specimens from oligotrophic waters of Japan Sea (Hokkaido)
Source: PLoS One. 2017 Jul 12;12(7):e0180760. doi: 10.1371/journal.pone.0180760 (PMC5507519; doi:10.1371/journal.pone.0180760)
Supplement: S6 Fig — In incubation experiments, one block (30×30×30 cm) was fertilized in a panlite tank and seawater adjusted to 15°C added at a rate of 200 L day-1. DIN concentrations in sea water using a panlite tank and δ15N changes in Saccharina japonica var. religiosa were determined. (PDF) [file pone.0180760.s006.pdf]

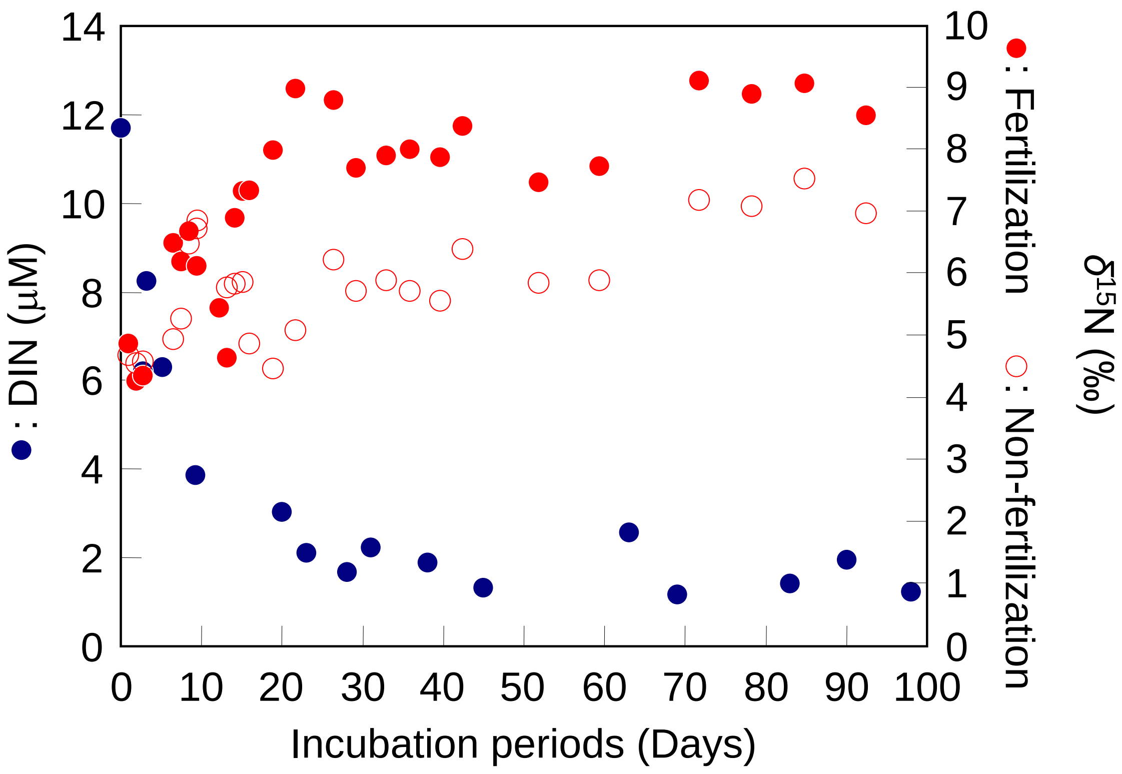

S6 Fig. DIN variations and $\delta^{15}N$ changes in *Saccharina japonica* var. *religiosa* in fertilization and non-fertilization in relation to incubation periods. In incubation experiments, one block (30×30×30 cm) was fertilized in a panlite tank and seawater adjusted to 15 °C added at a rate of 200 L day^{-1}. DIN concentrations in sea water using a panlite tank and $\delta^{15}N$ changes in *Saccharina japonica* var. *religiosa* were determined.
