# Supplementary material for: Historical δ15N records of Saccharina specimens from oligotrophic waters of Japan Sea (Hokkaido)
Source: PLoS One. 2017 Jul 12;12(7):e0180760. doi: 10.1371/journal.pone.0180760 (PMC5507519; doi:10.1371/journal.pone.0180760)
Supplement: S1 Table — (PDF) [file pone.0180760.s010.pdf]

S1 Table. δ^{15}N values (1881-2014) in *Saccharina* and other species of seaweeds specimens collected from coastal areas around Hokkaido, Japan.

Coastal areas around Hokkaido	Species	Year	δ^{15}N (‰)
Japan Sea	*Saccharina japonica*	1933	5.7
Japan Sea	*Saccharina japonica*	1988	7.2
Japan Sea	*Saccharina japonica*	1988	4.5
Japan Sea	*Saccharina japonica*	1992	4.5
Japan Sea	*Saccharina japonica*	1995	6.0
Japan Sea	*Saccharina japonica*	2005	5.3
Japan Sea	*Saccharina japonica* var. *ochotensis*	1891	8.5
Japan Sea	*Saccharina japonica* var. *ochotensis*	1891	9.1
Japan Sea	*Saccharina japonica* var. *ochotensis*	1891	9.7
Japan Sea	*Saccharina japonica* var. *ochotensis*	1891	11.3
Japan Sea	*Saccharina japonica* var. *ochotensis*	1891	10.1
Japan Sea	*Saccharina japonica* var. *ochotensis*	1892	10.4
Japan Sea	*Saccharina japonica* var. *ochotensis*	1892	10.8
Japan Sea	*Saccharina japonica* var. *ochotensis*	1892	10.4
Japan Sea	*Saccharina japonica* var. *ochotensis*	1894	11.1
Japan Sea	*Saccharina japonica* var. *ochotensis*	1894	9.9
Japan Sea	*Saccharina japonica* var. *ochotensis*	1894	10.3
Japan Sea	*Saccharina japonica* var. *ochotensis*	1894	9.8
Japan Sea	*Saccharina japonica* var. *ochotensis*	1894	10.0
Japan Sea	*Saccharina japonica* var. *ochotensis*	1894	9.1
Japan Sea	*Saccharina japonica* var. *ochotensis*	1896	11.5
Japan Sea	*Saccharina japonica* var. *ochotensis*	1896	10.0
Japan Sea	*Saccharina japonica* var. *ochotensis*	1928	9.7
Japan Sea	*Saccharina japonica* var. *ochotensis*	1929	10.6
Japan Sea	*Saccharina japonica* var. *ochotensis*	1929	9.3
Japan Sea	*Saccharina japonica* var. *ochotensis*	1932	9.2
Japan Sea	*Saccharina japonica* var. *ochotensis*	1932	9.1
Japan Sea	*Saccharina japonica* var. *ochotensis*	1932	8.3
Japan Sea	*Saccharina japonica* var. *ochotensis*	1954	5.7
Japan Sea	*Saccharina japonica* var. *ochotensis*	1958	6.2
Japan Sea	*Saccharina japonica* var. *ochotensis*	1970	5.1
Japan Sea	*Saccharina japonica* var. *ochotensis*	1980	7.1
Japan Sea	*Saccharina japonica* var. *ochotensis*	1980	6.5
Japan Sea	*Saccharina japonica* var. *ochotensis*	2005	5.2
Japan Sea	*Saccharina japonica* var. *ochotensis*	2005	5.7
Japan Sea	*Saccharina japonica* var. *ochotensis*	2010	7.2
Japan Sea	*Saccharina japonica* var. *ochotensis*	2010	4.3
Japan Sea	*Saccharina japonica* var. *ochotensis*	2011	3.8
Japan Sea	*Saccharina japonica* var. *ochotensis*	2011	6.6
Japan Sea	*Saccharina japonica* var. *ochotensis*	2011	4.0
Japan Sea	*Saccharina japonica* var. *ochotensis*	2011	5.2
Japan Sea	*Saccharina japonica* var. *ochotensis*	2011	7.0
Japan Sea	*Saccharina japonica* var. *ochotensis*	2011	5.4
Japan Sea	*Saccharina japonica* var. *ochotensis*	2011	7.6
Japan Sea	*Saccharina japonica* var. *ochotensis*	2011	4.9
Japan Sea	*Saccharina japonica* var. *ochotensis*	2011	5.3
Japan Sea	*Saccharina japonica* var. *ochotensis*	2011	5.4
Japan Sea	*Saccharina japonica* var. *ochotensis*	2011	7.6
Japan Sea	*Saccharina japonica* var. *ochotensis*	2011	5.8
Japan Sea	*Saccharina japonica* var. *ochotensis*	2011	4.5
Japan Sea	*Saccharina japonica* var. *ochotensis*	2014	6.3
Japan Sea	*Saccharina japonica* var. *ochotensis*	2014	6.6
Japan Sea	*Saccharina japonica* var. *ochotensis*	2014	6.6
Japan Sea	*Saccharina japonica* var. *religiosa*	1884	13.6
Japan Sea	*Saccharina japonica* var. *religiosa*	1887	12.6
Japan Sea	*Saccharina japonica* var. *religiosa*	1890	11.7
Japan Sea	*Saccharina japonica* var. *religiosa*	1891	10.6
Japan Sea	*Saccharina japonica* var. *religiosa*	1891	10.7
Japan Sea	*Saccharina japonica* var. *religiosa*	1891	7.3
Japan Sea	*Saccharina japonica* var. *religiosa*	1891	9.7
Japan Sea	*Saccharina japonica* var. *religiosa*	1893	9.3
Japan Sea	*Saccharina japonica* var. *religiosa*	1893	10.7
Japan Sea	*Saccharina japonica* var. *religiosa*	1894	9.5
Japan Sea	*Saccharina japonica* var. *religiosa*	1894	9.9
Japan Sea	*Saccharina japonica* var. *religiosa*	1894	9.9

Japan Sea	*Saccharina japonica* var. *religiosa*	1894	10.1
Japan Sea	*Saccharina japonica* var. *religiosa*	1894	11.3
Japan Sea	*Saccharina japonica* var. *religiosa*	1894	9.8
Japan Sea	*Saccharina japonica* var. *religiosa*	1894	9.9
Japan Sea	*Saccharina japonica* var. *religiosa*	1894	9.5
Japan Sea	*Saccharina japonica* var. *religiosa*	1894	10.5
Japan Sea	*Saccharina japonica* var. *religiosa*	1901	11.1
Japan Sea	*Saccharina japonica* var. *religiosa*	1901	11.2
Japan Sea	*Saccharina japonica* var. *religiosa*	1901	11.2
Japan Sea	*Saccharina japonica* var. *religiosa*	1910	11.9
Japan Sea	*Saccharina japonica* var. *religiosa*	1926	8.1
Japan Sea	*Saccharina japonica* var. *religiosa*	1929	8.3
Japan Sea	*Saccharina japonica* var. *religiosa*	1929	9.7
Japan Sea	*Saccharina japonica* var. *religiosa*	1929	9.7
Japan Sea	*Saccharina japonica* var. *religiosa*	1929	9.7
Japan Sea	*Saccharina japonica* var. *religiosa*	1930	6.6
Japan Sea	*Saccharina japonica* var. *religiosa*	1930	9.4
Japan Sea	*Saccharina japonica* var. *religiosa*	1930	6.0
Japan Sea	*Saccharina japonica* var. *religiosa*	1930	5.7
Japan Sea	*Saccharina japonica* var. *religiosa*	1930	8.3
Japan Sea	*Saccharina japonica* var. *religiosa*	1931	5.3
Japan Sea	*Saccharina japonica* var. *religiosa*	1932	8.1
Japan Sea	*Saccharina japonica* var. *religiosa*	1932	8.1
Japan Sea	*Saccharina japonica* var. *religiosa*	1932	7.3
Japan Sea	*Saccharina japonica* var. *religiosa*	1932	8.5
Japan Sea	*Saccharina japonica* var. *religiosa*	1932	10.0
Japan Sea	*Saccharina japonica* var. *religiosa*	1932	8.2
Japan Sea	*Saccharina japonica* var. *religiosa*	1933	7.6
Japan Sea	*Saccharina japonica* var. *religiosa*	1933	9.0
Japan Sea	*Saccharina japonica* var. *religiosa*	1933	8.8
Japan Sea	*Saccharina japonica* var. *religiosa*	1933	9.1
Japan Sea	*Saccharina japonica* var. *religiosa*	1933	6.7
Japan Sea	*Saccharina japonica* var. *religiosa*	1943	6.2
Japan Sea	*Saccharina japonica* var. *religiosa*	1943	6.3
Japan Sea	*Saccharina japonica* var. *religiosa*	1943	7.2
Japan Sea	*Saccharina japonica* var. *religiosa*	1943	5.6
Japan Sea	*Saccharina japonica* var. *religiosa*	1944	6.7
Japan Sea	*Saccharina japonica* var. *religiosa*	1944	10.5
Japan Sea	*Saccharina japonica* var. *religiosa*	1944	10.3
Japan Sea	*Saccharina japonica* var. *religiosa*	1946	7.4
Japan Sea	*Saccharina japonica* var. *religiosa*	1946	11.6
Japan Sea	*Saccharina japonica* var. *religiosa*	1948	7.2
Japan Sea	*Saccharina japonica* var. *religiosa*	1954	6.1
Japan Sea	*Saccharina japonica* var. *religiosa*	1983	8.2
Japan Sea	*Saccharina japonica* var. *religiosa*	1984	6.4
Japan Sea	*Saccharina japonica* var. *religiosa*	1984	6.6
Japan Sea	*Saccharina japonica* var. *religiosa*	1984	6.4
Japan Sea	*Saccharina japonica* var. *religiosa*	1985	6.6
Japan Sea	*Saccharina japonica* var. *religiosa*	1985	6.9
Japan Sea	*Saccharina japonica* var. *religiosa*	1985	4.2
Japan Sea	*Saccharina japonica* var. *religiosa*	1985	4.0
Japan Sea	*Saccharina japonica* var. *religiosa*	1987	6.8
Japan Sea	*Saccharina japonica* var. *religiosa*	1988	6.0
Japan Sea	*Saccharina japonica* var. *religiosa*	1989	5.2
Japan Sea	*Saccharina japonica* var. *religiosa*	1991	6.2
Japan Sea	*Saccharina japonica* var. *religiosa*	1995	7.1
Japan Sea	*Saccharina japonica* var. *religiosa*	1995	7.8
Japan Sea	*Saccharina japonica* var. *religiosa*	1995	7.3
Japan Sea	*Saccharina japonica* var. *religiosa*	1995	7.4
Japan Sea	*Saccharina japonica* var. *religiosa*	1997	6.8
Japan Sea	*Saccharina japonica* var. *religiosa*	1997	2.9
Japan Sea	*Saccharina japonica* var. *religiosa*	1997	5.6
Japan Sea	*Saccharina japonica* var. *religiosa*	2005	5.3
Japan Sea	*Saccharina japonica* var. *religiosa*	2005	5.2
Japan Sea	*Saccharina japonica* var. *religiosa*	2009	5.5
Japan Sea	*Saccharina japonica* var. *religiosa*	2009	5.1
Japan Sea	*Saccharina japonica* var. *religiosa*	2009	6.0
Japan Sea	*Saccharina japonica* var. *religiosa*	2009	2.6
Japan Sea	*Saccharina japonica* var. *religiosa*	2010	6.5
Japan Sea	*Saccharina japonica* var. *religiosa*	2010	5.7
Japan Sea	*Saccharina japonica* var. *religiosa*	2010	2.4

Japan Sea	*Saccharina japonica* var. *religiosa*	2010	2.6
Japan Sea	*Saccharina japonica* var. *religiosa*	2010	2.4
Japan Sea	*Saccharina japonica* var. *religiosa*	2010	2.1
Japan Sea	*Saccharina japonica* var. *religiosa*	2010	6.6
Japan Sea	*Saccharina japonica* var. *religiosa*	2010	3.1
Japan Sea	*Saccharina japonica* var. *religiosa*	2010	2.4
Japan Sea	*Saccharina japonica* var. *religiosa*	2011	6.4
Japan Sea	*Saccharina japonica* var. *religiosa*	2011	3.7
Japan Sea	*Saccharina japonica* var. *religiosa*	2011	4.8
Japan Sea	*Saccharina japonica* var. *religiosa*	2011	6.9
Japan Sea	*Saccharina japonica* var. *religiosa*	2011	6.2
Japan Sea	*Saccharina japonica* var. *religiosa*	2011	6.6
Japan Sea	*Saccharina japonica* var. *religiosa*	2011	4.5
Japan Sea	*Saccharina japonica* var. *religiosa*	2011	5.3
Japan Sea	*Saccharina japonica* var. *religiosa*	2011	4.4
Japan Sea	*Saccharina japonica* var. *religiosa*	2011	4.2
Japan Sea	*Saccharina japonica* var. *religiosa*	2011	3.9
Japan Sea	*Saccharina japonica* var. *religiosa*	2011	3.2
Japan Sea	*Saccharina japonica* var. *religiosa*	2011	3.3
Japan Sea	*Saccharina japonica* var. *religiosa*	2011	6.6
Japan Sea	*Saccharina japonica* var. *religiosa*	2011	3.5
Japan Sea	*Saccharina japonica* var. *religiosa*	2011	4.8
Japan Sea	*Saccharina japonica* var. *religiosa*	2011	4.0
Japan Sea	*Saccharina japonica* var. *religiosa*	2011	5.3
Japan Sea	*Saccharina japonica* var. *religiosa*	2011	5.6
Japan Sea	*Saccharina japonica* var. *religiosa*	2011	4.9
Japan Sea	*Saccharina japonica* var. *religiosa*	2011	5.0
Japan Sea	*Saccharina japonica* var. *religiosa*	2011	4.9
Japan Sea	*Saccharina japonica* var. *religiosa*	2011	6.2
Japan Sea	*Saccharina japonica* var. *religiosa*	2011	4.7
Japan Sea	*Saccharina japonica* var. *religiosa*	2011	4.7
Japan Sea	*Saccharina japonica* var. *religiosa*	2011	5.2
Japan Sea	*Saccharina japonica* var. *religiosa*	2011	2.8
Japan Sea	*Saccharina japonica* var. *religiosa*	2011	4.2
Japan Sea	*Saccharina japonica* var. *religiosa*	2011	4.4
Japan Sea	*Saccharina japonica* var. *religiosa*	2011	3.0
Japan Sea	*Saccharina japonica* var. *religiosa*	2011	5.9
Japan Sea	*Saccharina japonica* var. *religiosa*	2011	3.6
Japan Sea	*Saccharina japonica* var. *religiosa*	2011	3.1
Japan Sea	*Saccharina japonica* var. *religiosa*	2011	3.9
Japan Sea	*Saccharina japonica* var. *religiosa*	2011	4.9
Japan Sea	*Saccharina japonica* var. *religiosa*	2011	4.9
Japan Sea	*Saccharina japonica* var. *religiosa*	2011	4.5
Japan Sea	*Saccharina japonica* var. *religiosa*	2011	4.3
Japan Sea	*Saccharina japonica* var. *religiosa*	2011	6.1
Japan Sea	*Saccharina japonica* var. *religiosa*	2011	6.4
Japan Sea	*Saccharina japonica* var. *religiosa*	2011	6.6
Japan Sea	*Saccharina japonica* var. *religiosa*	2011	3.6
Japan Sea	*Saccharina japonica* var. *religiosa*	2012	5.7
Japan Sea	*Saccharina japonica* var. *religiosa*	2012	6.9
Okhotsk Sea	*Saccharina japonica* var. *diabolica*	1894	4.4
Okhotsk Sea	*Saccharina japonica* var. *diabolica*	1931	5.9
Okhotsk Sea	*Saccharina japonica* var. *diabolica*	1931	4.0
Okhotsk Sea	*Saccharina japonica* var. *diabolica*	1931	4.4
Okhotsk Sea	*Saccharina japonica* var. *diabolica*	1931	6.8
Okhotsk Sea	*Saccharina japonica* var. *diabolica*	1968	3.0
Okhotsk Sea	*Saccharina japonica* var. *diabolica*	1970	2.5
Okhotsk Sea	*Saccharina japonica* var. *diabolica*	1987	2.9
Okhotsk Sea	*Saccharina japonica* var. *diabolica*	2010	3.7
Okhotsk Sea	*Saccharina japonica* var. *diabolica*	2010	5.6
Okhotsk Sea	*Saccharina japonica* var. *diabolica*	2011	5.0
Okhotsk Sea	*Saccharina japonica* var. *diabolica*	2011	9.5
Okhotsk Sea	*Saccharina japonica* var. *ochotensis*	1894	5.5
Okhotsk Sea	*Saccharina japonica* var. *ochotensis*	1931	5.4
Okhotsk Sea	*Saccharina japonica* var. *ochotensis*	1933	6.2
Okhotsk Sea	*Saccharina japonica* var. *ochotensis*	2014	4.4
Okhotsk Sea	*Saccharina japonica* var. *ochotensis*	2014	4.0
Okhotsk Sea	*Saccharina longissima*	1894	4.1
Okhotsk Sea	*Saccharina longissima*	2011	8.9
Okhotsk Sea	*Saccharina longissima*	2011	7.9
Pacific Ocean	*Saccharina angustata*	1890	6.8

Pacific Ocean	*Saccharina angustata*	1890	8.3
Pacific Ocean	*Saccharina angustata*	1890	6.6
Pacific Ocean	*Saccharina angustata*	1891	4.5
Pacific Ocean	*Saccharina angustata*	1892	7.7
Pacific Ocean	*Saccharina angustata*	1892	7.6
Pacific Ocean	*Saccharina angustata*	1893	7.4
Pacific Ocean	*Saccharina angustata*	1894	7.0
Pacific Ocean	*Saccharina angustata*	1894	6.9
Pacific Ocean	*Saccharina angustata*	1894	8.5
Pacific Ocean	*Saccharina angustata*	1894	7.4
Pacific Ocean	*Saccharina angustata*	1894	6.8
Pacific Ocean	*Saccharina angustata*	1894	6.3
Pacific Ocean	*Saccharina angustata*	1894	7.0
Pacific Ocean	*Saccharina angustata*	1894	6.4
Pacific Ocean	*Saccharina angustata*	1894	6.7
Pacific Ocean	*Saccharina angustata*	1894	6.9
Pacific Ocean	*Saccharina angustata*	1894	6.6
Pacific Ocean	*Saccharina angustata*	1930	7.9
Pacific Ocean	*Saccharina angustata*	1931	7.3
Pacific Ocean	*Saccharina angustata*	1931	8.3
Pacific Ocean	*Saccharina angustata*	1931	8.0
Pacific Ocean	*Saccharina angustata*	1933	3.6
Pacific Ocean	*Saccharina angustata*	1934	5.8
Pacific Ocean	*Saccharina angustata*	1942	7.8
Pacific Ocean	*Saccharina angustata*	1948	5.9
Pacific Ocean	*Saccharina angustata*	1953	10.6
Pacific Ocean	*Saccharina angustata*	1955	8.1
Pacific Ocean	*Saccharina angustata*	1955	8.5
Pacific Ocean	*Saccharina angustata*	1956	7.2
Pacific Ocean	*Saccharina angustata*	1963	10.3
Pacific Ocean	*Saccharina angustata*	1966	5.2
Pacific Ocean	*Saccharina angustata*	1975	7.8
Pacific Ocean	*Saccharina angustata*	1985	7.2
Pacific Ocean	*Saccharina angustata*	1996	7.4
Pacific Ocean	*Saccharina angustata*	2010	6.6
Pacific Ocean	*Saccharina angustata*	2011	7.1
Pacific Ocean	*Saccharina angustata*	2014	5.9
Pacific Ocean	*Saccharina angustata*	2014	6.0
Pacific Ocean	*Saccharina angustata*	2014	6.6
Pacific Ocean	*Saccharina angustata*	2014	6.4
Pacific Ocean	*Saccharina angustata*	2014	5.9
Pacific Ocean	*Saccharina coriacea*	1956	9.5
Pacific Ocean	*Saccharina coriacea*	1956	6.1
Pacific Ocean	*Saccharina coriacea*	1987	6.3
Pacific Ocean	*Saccharina coriacea*	1993	4.9
Pacific Ocean	*Saccharina coriacea*	1993	4.3
Pacific Ocean	*Saccharina coriacea*	1996	9.5
Pacific Ocean	*Saccharina coriacea*	2010	7.0
Pacific Ocean	*Saccharina coriacea*	2011	6.3
Pacific Ocean	*Saccharina coriacea*	2011	6.5
Pacific Ocean	*Saccharina coriacea*	2014	4.4
Pacific Ocean	*Saccharina coriacea*	2014	6.1
Pacific Ocean	*Saccharina coriacea*	2014	6.8
Pacific Ocean	*Saccharina japonica*	1890	6.0
Pacific Ocean	*Saccharina japonica*	1890	3.6
Pacific Ocean	*Saccharina japonica*	1890	7.3
Pacific Ocean	*Saccharina japonica*	1890	6.8
Pacific Ocean	*Saccharina japonica*	1890	7.6
Pacific Ocean	*Saccharina japonica*	1890	7.2
Pacific Ocean	*Saccharina japonica*	1890	7.5
Pacific Ocean	*Saccharina japonica*	1890	7.3
Pacific Ocean	*Saccharina japonica*	1890	6.5
Pacific Ocean	*Saccharina japonica*	1891	6.7
Pacific Ocean	*Saccharina japonica*	1894	11.4
Pacific Ocean	*Saccharina japonica*	1894	11.6
Pacific Ocean	*Saccharina japonica*	1894	5.3
Pacific Ocean	*Saccharina japonica*	1894	6.6
Pacific Ocean	*Saccharina japonica*	1930	5.7
Pacific Ocean	*Saccharina japonica*	1931	6.7
Pacific Ocean	*Saccharina japonica*	1931	6.2
Pacific Ocean	*Saccharina japonica*	1931	7.2

Pacific Ocean	*Saccharina japonica*	1931	7.2
Pacific Ocean	*Saccharina japonica*	1931	7.1
Pacific Ocean	*Saccharina japonica*	1931	6.9
Pacific Ocean	*Saccharina japonica*	1931	6.5
Pacific Ocean	*Saccharina japonica*	1933	7.5
Pacific Ocean	*Saccharina japonica*	1934	7.7
Pacific Ocean	*Saccharina japonica*	1935	9.2
Pacific Ocean	*Saccharina japonica*	1936	2.7
Pacific Ocean	*Saccharina japonica*	1936	9.1
Pacific Ocean	*Saccharina japonica*	1972	6.3
Pacific Ocean	*Saccharina japonica*	1973	8.0
Pacific Ocean	*Saccharina japonica*	1985	6.0
Pacific Ocean	*Saccharina japonica*	1988	6.8
Pacific Ocean	*Saccharina japonica*	1998	6.2
Pacific Ocean	*Saccharina japonica*	2003	7.4
Pacific Ocean	*Saccharina japonica*	2010	5.2
Pacific Ocean	*Saccharina japonica*	2010	4.0
Pacific Ocean	*Saccharina japonica*	2011	5.2
Pacific Ocean	*Saccharina japonica*	2011	5.0
Pacific Ocean	*Saccharina japonica*	2011	5.2
Pacific Ocean	*Saccharina japonica*	2014	4.1
Pacific Ocean	*Saccharina japonica*	2014	5.5
Pacific Ocean	*Saccharina japonica*	2014	5.8
Pacific Ocean	*Saccharina japonica*	2014	6.0
Pacific Ocean	*Saccharina japonica*	2014	6.1
Pacific Ocean	*Saccharina japonica*	2014	6.3
Pacific Ocean	*Saccharina japonica*	2014	6.4
Pacific Ocean	*Saccharina japonica*	2014	6.5
Pacific Ocean	*Saccharina japonica*	2014	6.7
Pacific Ocean	*Saccharina japonica*	2014	6.7
Pacific Ocean	*Saccharina japonica*	2014	6.8
Pacific Ocean	*Saccharina japonica*	2014	7.1
Pacific Ocean	*Saccharina japonica*	2014	7.2
Pacific Ocean	*Saccharina japonica*	2014	8.8
Pacific Ocean	*Saccharina japonica*	2014	8.8
Pacific Ocean	*Saccharina japonica* var. *diabolica*	1892	8.1
Pacific Ocean	*Saccharina japonica* var. *diabolica*	1930	4.3
Pacific Ocean	*Saccharina japonica* var. *diabolica*	1959	3.0
Pacific Ocean	*Saccharina japonica* var. *diabolica*	1959	2.7
Pacific Ocean	*Saccharina japonica* var. *religiosa*	1917	6.5
Pacific Ocean	*Saccharina japonica* var. *religiosa*	1917	5.5
Pacific Ocean	*Saccharina japonica* var. *religiosa*	1917	5.4
Pacific Ocean	*Saccharina japonica* var. *religiosa*	1917	6.1
Pacific Ocean	*Saccharina japonica* var. *religiosa*	1932	6.0
Pacific Ocean	*Saccharina japonica* var. *religiosa*	1933	5.8
Pacific Ocean	*Saccharina japonica* var. *religiosa*	1933	7.2
Pacific Ocean	*Saccharina japonica* var. *religiosa*	1936	10.9
Pacific Ocean	*Saccharina japonica* var. *religiosa*	1936	11.3
Pacific Ocean	*Saccharina japonica* var. *religiosa*	1943	6.4
Pacific Ocean	*Saccharina japonica* var. *religiosa*	1988	7.1
Pacific Ocean	*Saccharina japonica* var. *religiosa*	1988	4.6
Pacific Ocean	*Saccharina longissima*	1924	8.4
Pacific Ocean	*Saccharina longissima*	1933	9.5
Pacific Ocean	*Saccharina longissima*	1969	7.5
Pacific Ocean	*Saccharina longissima*	1987	8.0
Pacific Ocean	*Saccharina longissima*	1988	8.6
Pacific Ocean	*Saccharina longissima*	1988	6.7
Pacific Ocean	*Saccharina longissima*	1993	7.7
Pacific Ocean	*Saccharina longissima*	2010	8.4
Pacific Ocean	*Saccharina longissima*	2011	8.7
Pacific Ocean	*Saccharina longissima*	2011	8.1
Pacific Ocean	*Saccharina sculpera*	1890	4.9
Pacific Ocean	*Saccharina sculpera*	1933	7.6
Pacific Ocean	*Saccharina sculpera*	1933	7.1
Pacific Ocean	*Saccharina sculpera*	2010	5.8
Pacific Ocean	*Saccharina sculpera*	2011	3.7
Pacific Ocean	*Saccharina yendoana*	1933	7.2
Pacific Ocean	*Saccharina yendoana*	1933	6.5
Pacific Ocean	*Saccharina yendoana*	1933	8.3
Pacific Ocean	*Saccharina yendoana*	1966	8.7
Japan Sea	Other species of seaweeds	1881	11.4

Japan Sea	Other species of seaweeds	1884	9.7
Japan Sea	Other species of seaweeds	1889	8.4
Japan Sea	Other species of seaweeds	1890	9.2
Japan Sea	Other species of seaweeds	1890	12.6
Japan Sea	Other species of seaweeds	1891	13.8
Japan Sea	Other species of seaweeds	1894	9.8
Japan Sea	Other species of seaweeds	1899	14.4
Japan Sea	Other species of seaweeds	1901	7.4
Japan Sea	Other species of seaweeds	1901	8.3
Japan Sea	Other species of seaweeds	1910	12.3
Japan Sea	Other species of seaweeds	1929	7.9
Japan Sea	Other species of seaweeds	1929	8.0
Japan Sea	Other species of seaweeds	1929	8.9
Japan Sea	Other species of seaweeds	1929	7.2
Japan Sea	Other species of seaweeds	1929	8.1
Japan Sea	Other species of seaweeds	1929	8.1
Japan Sea	Other species of seaweeds	1930	5.7
Japan Sea	Other species of seaweeds	1933	6.2
Japan Sea	Other species of seaweeds	1933	5.2
Japan Sea	Other species of seaweeds	1933	6.2
Japan Sea	Other species of seaweeds	1933	5.1
Japan Sea	Other species of seaweeds	1943	6.5
Japan Sea	Other species of seaweeds	1943	6.6
Japan Sea	Other species of seaweeds	1950	5.1
Japan Sea	Other species of seaweeds	1953	7.5
Japan Sea	Other species of seaweeds	1965	7.9
Japan Sea	Other species of seaweeds	1988	3.6
Japan Sea	Other species of seaweeds	1988	2.8
Japan Sea	Other species of seaweeds	2009	6.4
Japan Sea	Other species of seaweeds	2009	5.7
Japan Sea	Other species of seaweeds	2009	5.0
Japan Sea	Other species of seaweeds	2009	7.1
Japan Sea	Other species of seaweeds	2009	6.3
Japan Sea	Other species of seaweeds	2009	5.4
Japan Sea	Other species of seaweeds	2009	4.6
Japan Sea	Other species of seaweeds	2010	4.4
Japan Sea	Other species of seaweeds	2010	5.2
Japan Sea	Other species of seaweeds	2010	3.1
Japan Sea	Other species of seaweeds	2010	3.0
Japan Sea	Other species of seaweeds	2010	5.1
Japan Sea	Other species of seaweeds	2010	5.0
Japan Sea	Other species of seaweeds	2010	4.1
Japan Sea	Other species of seaweeds	2010	2.7
Japan Sea	Other species of seaweeds	2010	4.6
Japan Sea	Other species of seaweeds	2010	3.6
Japan Sea	Other species of seaweeds	2010	4.9
Japan Sea	Other species of seaweeds	2010	2.6
Japan Sea	Other species of seaweeds	2010	3.7
Japan Sea	Other species of seaweeds	2010	3.3
Japan Sea	Other species of seaweeds	2010	5.8
Japan Sea	Other species of seaweeds	2010	5.2
Japan Sea	Other species of seaweeds	2010	4.7
Japan Sea	Other species of seaweeds	2010	5.4
Japan Sea	Other species of seaweeds	2010	4.6
Japan Sea	Other species of seaweeds	2010	5.9
Japan Sea	Other species of seaweeds	2010	6.0
Japan Sea	Other species of seaweeds	2010	3.1
Japan Sea	Other species of seaweeds	2010	4.3
Japan Sea	Other species of seaweeds	2010	6.2
Japan Sea	Other species of seaweeds	2010	5.4
Japan Sea	Other species of seaweeds	2010	4.0
Japan Sea	Other species of seaweeds	2010	4.6
Japan Sea	Other species of seaweeds	2010	4.5
Japan Sea	Other species of seaweeds	2010	3.7
Japan Sea	Other species of seaweeds	2011	6.8
Japan Sea	Other species of seaweeds	2011	4.1
Japan Sea	Other species of seaweeds	2011	4.9
Japan Sea	Other species of seaweeds	2011	4.1
Japan Sea	Other species of seaweeds	2011	2.6
