# Supplementary material for: Historical δ15N records of Saccharina specimens from oligotrophic waters of Japan Sea (Hokkaido)
Source: PLoS One. 2017 Jul 12;12(7):e0180760. doi: 10.1371/journal.pone.0180760 (PMC5507519; doi:10.1371/journal.pone.0180760)
Supplement: S2 Table — (PDF) [file pone.0180760.s011.pdf]

**S2 Table. $\delta^{15}N$ values of herring sperm, eggs, and herring processing residue.**

| Types | $\delta^{15}N$ (‰) |
|---|---|
| Sperm1 | 8.7 |
| Sperm2 | 9.1 |
| Sperm3 | 8.7 |
| Sperm4 | 8.7 |
| Sperm5 | 9.6 |
| Sperm6 | 9.5 |
| Sperm7 | 9.3 |
| Sperm8 | 9.4 |
| Sperm9 | 9.1 |
| Sperm10 | 9.2 |
| Egg1 | 10.9 |
| Egg2 | 10.8 |
| Egg3 | 10.8 |
| Egg4 | 10.2 |
| Egg5 | 10.3 |
| Egg6 | 11.0 |
| Egg7 | 10.9 |
| Egg8 | 10.6 |
| Egg9 | 11.4 |
| Egg10 | 11.2 |
| Processing residues1 | 11.3 |
| Processing residues2 | 12.0 |
| Processing residues3 | 10.4 |
| Processing residues4 | 10.9 |
| Processing residues5 | 12.4 |
| Processing residues6 | 11.9 |
| Processing residues7 | 11.7 |
| Processing residues8 | 13.3 |
| Processing residues9 | 11.7 |
| Processing residues10 | 13.8 |
