# Supplementary material for: Historical δ15N records of Saccharina specimens from oligotrophic waters of Japan Sea (Hokkaido)
Source: PLoS One. 2017 Jul 12;12(7):e0180760. doi: 10.1371/journal.pone.0180760 (PMC5507519; doi:10.1371/journal.pone.0180760)
Supplement: S3 Table — δ15N in seaweeds on the coast off Otaru (Shukutsu and Asari) were high in 9 March 2013 after “Kuki”. (PDF) [file pone.0180760.s012.pdf]

S3 Table. DIN and PON concentration, δ^{15}N-PON values, and δ^{15}N in seaweeds on the coast off Otaru (Shukutsu) and control site (Yoichi) on 8 February 2013, Otaru (Asari) and control site (Yoichi) on 14 February 2013 when male herring spawning caused the appearance of milky-white turbidity at the sea surface, called "*Kuki*" though it was a small-scale event compared with the past. δ^{15}N in seaweeds on the coast off Otaru (Shukutsu and Asari) were high in 9 March 2013 after "*Kuki*".

Date	Site	DIN (μM)	PON (μM)	δ^{15}N-PON (‰)	δ^{15}N in seaweeds (‰)	
					8 February 2013	9 March 2013
8 February 2013	Otaru (Shukutsu)	64.7	73.3	11.3	4.4	9.2
		95.8	72.3	11.2	3.7	7.4
		40.0	61.8	11.1	5.7	8.5
	Control (Yoichi)	5.0	1.4	4.9	4.0	3.8
		4.9	1.2	4.3	5.0	3.7

Date	Site	DIN (μM)	PON (μM)	δ^{15}N-PON (‰)	δ^{15}N in seaweeds (‰)	
					14 February 2013	9 March 2013
14 February 2013	Otaru (Asari)	37.3	95.2	10.8	6.3	6.8
		33.9	83.6	11.1	6.8	8.9
		49.1	86.1	11.5	4.5	9.6
	Control (Yoichi)	4.5	1.6	4.1	3.4	5.4
		4.3	1.3	4.3	5.8	5.2
