# Supplementary material for: Historical δ15N records of Saccharina specimens from oligotrophic waters of Japan Sea (Hokkaido)
Source: PLoS One. 2017 Jul 12;12(7):e0180760. doi: 10.1371/journal.pone.0180760 (PMC5507519; doi:10.1371/journal.pone.0180760)
Supplement: S4 Table — (PDF) [file pone.0180760.s013.pdf]

S4 Table. δ^{15}N values in algal tissues of *Saccharina japonica* var. *religiosa* before and after fertilization at the fertilization point, non-fertilization point, and the fertilizer made from fish processing residue with wood chip ("Fertilizer").

Periods	Points	No.	δ^{15}N (‰)
Before fertilizing		1	3.1
Before fertilizing	Fertilization point	2	4.2
Before fertilizing	Fertilization point	3	4.2
Before fertilizing	Fertilization point	4	4.1
Before fertilizing	Fertilization point	5	5.7
Before fertilizing	Fertilization point	6	4.4
Before fertilizing	Fertilization point	7	6.6
Before fertilizing	Fertilization point	8	6.7
Before fertilizing	Fertilization point	9	5.6
Before fertilizing	Fertilization point	10	5.4
Before fertilizing	Fertilization point	11	3.0
Before fertilizing	Fertilization point	12	5.3
Before fertilizing	Non-fertilization point	1	6.1
Before fertilizing	Non-fertilization point	2	4.7
Before fertilizing	Non-fertilization point	3	4.8
Before fertilizing	Non-fertilization point	4	4.8
Before fertilizing	Non-fertilization point	5	3.8
Before fertilizing	Non-fertilization point	6	6.6
After fertilizing	Fertilization point	1	11.0
After fertilizing	Fertilization point	2	11.4
After fertilizing	Fertilization point	3	12.0
After fertilizing	Fertilization point	4	12.1
After fertilizing	Fertilization point	5	11.6
After fertilizing	Fertilization point	6	10.4
After fertilizing	Fertilization point	7	10.8
After fertilizing	Fertilization point	8	10.7
After fertilizing	Fertilization point	9	10.8
After fertilizing	Fertilization point	10	11.5
After fertilizing	Fertilization point	11	10.5
After fertilizing	Fertilization point	12	11.2
After fertilizing	Fertilization point	13	10.4
After fertilizing	Fertilization point	14	8.3
After fertilizing	Fertilization point	15	9.6
After fertilizing	Fertilization point	16	8.5
After fertilizing	Fertilization point	17	7.8
After fertilizing	Fertilization point	18	9.7
After fertilizing	Fertilization point	19	7.2
After fertilizing	Non-fertilization point	1	4.6
After fertilizing	Non-fertilization point	2	4.2
After fertilizing	Non-fertilization point	3	3.0
After fertilizing	Non-fertilization point	4	3.3
After fertilizing	Non-fertilization point	5	5.3
After fertilizing	Non-fertilization point	6	5.6
	Fertilizer	1	12.0
	Fertilizer	2	12.9
	Fertilizer	3	12.2
	Fertilizer	4	12.6
	Fertilizer	5	11.3
