# Supplementary material for: Historical δ15N records of Saccharina specimens from oligotrophic waters of Japan Sea (Hokkaido)
Source: PLoS One. 2017 Jul 12;12(7):e0180760. doi: 10.1371/journal.pone.0180760 (PMC5507519; doi:10.1371/journal.pone.0180760)
Supplement: S8 Fig — (PDF) [file pone.0180760.s008.pdf]

22 May 2017

Dr. Takanori Kuribayashi
Hokkaido Nuclear Energy Environmental Research Center

Dear Dr. Kuribayashi,

I grant explicitly permission for the open-access journal PLOS ONE to publish "Historical $\delta^{15}N$ records of *Saccharina* specimens from oligotrophic waters of Japan Sea (Hokkaido)" under a CC BY license.

Sincerely yours,

Shoichi Akaike

Shoichi Akaike
Local Independent Administrative Agency,
Hokkaido Research Organization, Fisheries Research Department,
Hamanaka-cho, Yoichi, Hokkaido 046-8555, Japan

S8 Fig. The granted permission from the copyright holder to publish Figure 3c under a CC BY license.
